# BN‐Doped Metal–Organic Frameworks: Tailoring 2D and 3D Porous Architectures through Molecular Editing of Borazines

**DOI:** 10.1002/chem.202004640

**Published:** 2021-01-31

**Authors:** Francesco Fasano, Jacopo Dosso, C. Grazia Bezzu, Mariolino Carta, François Kerff, Nicola Demitri, Bao‐Lian Su, Davide Bonifazi

**Affiliations:** ^1^ School of Chemistry Cardiff University Park Place Cardiff CF10 3AT UK; ^2^ Department of Chemistry Swansea University Grove Building, Singleton Park Swansea SA28PP UK; ^3^ Elettra—Sincrotrone Trieste S.S. 14 Km 163.5 in Area Science Park 34149 Basovizza Trieste Italy; ^4^ Namur Institute of Structured Matter (NISM) University of Namur 61 rue de Bruxelles 5000 Namur Belgium; ^5^ Institute of Organic Chemistry, Faculty of Chemistry University of Vienna Währinger Strasse 38 1090 Vienna Austria

**Keywords:** BN-doping, borazine, gas adsorption, heteroatom doping, meta–organic frameworks

## Abstract

Building on the MOF approach to prepare porous materials, herein we report the engineering of porous BN‐doped materials using tricarboxylic hexaarylborazine ligands, which are laterally decorated with functional groups at the full‐carbon ‘inner shell’. Whilst an open porous 3D entangled structure could be obtained from the double interpenetration of two identical metal frameworks derived from the methyl substituted borazine, the chlorine‐functionalised linker undergoes formation of a porous layered 2D honeycomb structure, as shown by single‐crystal X‐ray diffraction analysis. In this architecture, the borazine cores are rotated by 60° in alternating layers, thus generating large rhombohedral channels running perpendicular to the planes of the networks. An analogous unsubstituted full‐carbon metal framework was synthesised for comparison. The resulting MOF revealed a crystalline 3D entangled porous structure, composed by three mutually interpenetrating networks, hence denser than those obtained from the borazine linkers. Their microporosity and CO_2_ uptake were investigated, with the porous 3D BN‐MOF entangled structure exhibiting a large apparent BET specific surface area (1091 m^2^ g^−1^) and significant CO_2_ reversible adsorption (3.31 mmol g^−1^) at 1 bar and 273 K.

## Graphical abstract

**The borazine unit** was used to prepare crystalline porous MOFs from specifically designed borazine carboxylate linkers. To evaluate the influence of the borazine rings on the gas adsorption properties, the analogous full‐carbon carboxylate linker and the corresponding MOF were also prepared. The structure of the three MOFs was unambiguously determined by single crystal X‐ray analysis.

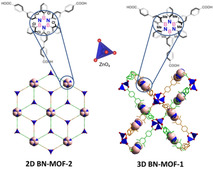

## Introduction

Over the last few decades environmental concerns such as climate change, mainly due to global warming caused by greenhouse gases, have stimulated the scientific community towards the research of clean energy carriers such as H_2_ along with the reduction of CO_2_ emissions generated from the combustion of fossil fuel. In this context the preparation of porous organic materials with predefined porosity displaying high surface areas has attracted unprecedented attention due to their great potentials for gas storage and separation.^[1]^ Porous organic polymers (POPs),^[2]^ porous molecular solids,^[3]^ covalent organic frameworks (COFs)^[4]^ and metal–organic frameworks (MOFs)^[5]^ have emerged in the last years as structures of choice to engineer microporous materials. The possibility to chemically edit the organic linkers allowed researchers in the field to customize the physical and chemical properties of such porous materials, producing an extraordinarily large number of functional architectures.^[6]^

Among the different functionalization strategies, the doping route, that is, the replacement of C atoms with isostructural species, is emerging as a versatile approach to tailor the physical and chemical properties of organic molecules.^[7]^ The replacement of C=C bonds by isostructural and isoelectronic polar B−N covalent couples considerably alters the character of the frontier molecular orbitals as well as the polarization properties of the π‐conjugated molecular surface.^[8]^ For instance, the introduction of polarized organic linkers within porous materials is very promising for enhancing interactions with polarized molecular species.[^5a^, ^9^] In this context, BN‐doped materials are good candidates for tailoring gas adsorption as the polar BN bonds could serve as active surface for the non‐covalent adsorption of gas molecules^[10]^ such as CO_2_.[^5a^, ^9^] Nanostructured boron nitrides,^[11]^ boron carbon nitrides,[^10a^, ^11b^, ^12^] BN‐decorated nanoporous carbons,^[10b]^ have been investigated for gas storage and shown high H_2_ and/or CO_2_ uptake. In particular, porous boron nitrides have been used in catalysis,^[13]^ CO_2_ capture,^[14]^ hydrogen storage[^10a^, ^15^] and water purification.^[16]^ Alternative porous materials containing BN bonds were reported by El‐Kaderi and co‐workers who proposed the use of borazine precursors to fabricate highly porous borazino polymers.^[17]^ These amorphous microporous polymers, showed a good CO_2_ uptake up to 3.18 mmol g^−1^ at 273 K, which has been ascribed to the interactions between the polarizable CO_2_ molecules and the borazine units. Taking into consideration these interactions, Xu and co‐workers, instead, proposed the use of MOFs as templates to obtain either porous BN‐containing MOF composite materials^[10c]^ or porous boron‐nitride‐carbides,^[10b]^ which showed significant CO_2_ adsorption (4.6 and 4.4 mmol g^−1^, respectively at 273 K). Although these represent interesting strategies to trigger the gas storage performance of MOFs, the preparation of BNC‐frameworks is limited by difficulties to control the BN grafting by MOF post‐synthetic modifications. A more promising method to form highly pure organised porous MOFs could be the use of organic linkers doped with BN functionalities. Boron imidazolate frameworks, which are formed by coordination of boron imidazolate linkers with metal ions, are an example of this type of materials.^[18]^ Inspired by these works, we anticipated that properly functionalised borazine molecules could serve as BN molecular ligands for engineering highly porous MOFs. Indeed, borazines have the perfect shape and functionalities to form trigonal three‐carboxylate linkers analogous to those used for making ultrahigh porous MOFs such as MOF‐177^[19]^ and MOF‐200,^[20]^ which are among those with the highest apparent BET specific surface area reported so far. Hence, herein we describe the synthesis, the single‐crystal X‐ray structures and gas uptake study of the first BN‐doped MOFs engineered with hexaryl‐substituted borazine carboxylate linkers.

## Results and Discussions

### Design and molecular editing of the borazine linkers

**Figure 1:**
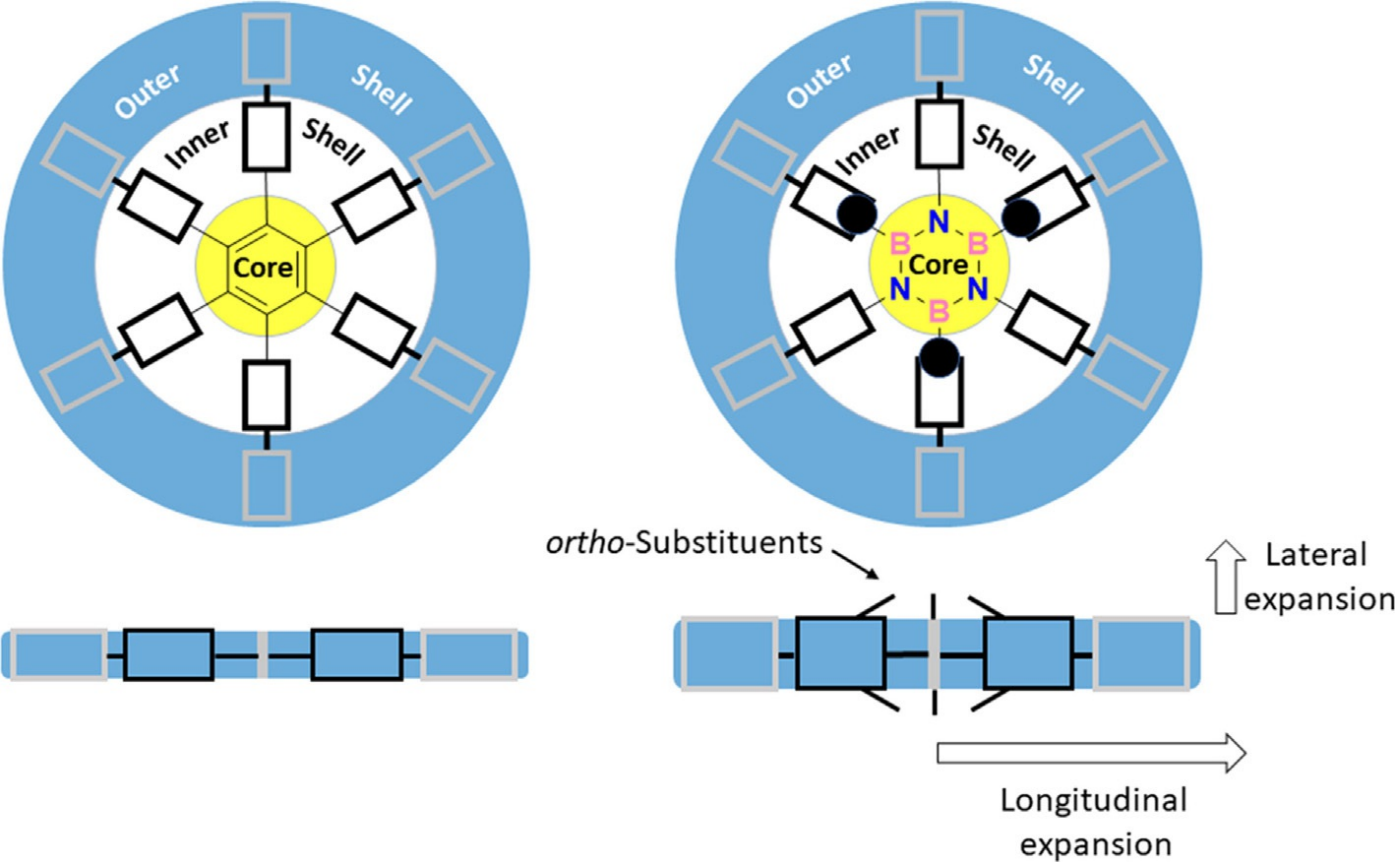
Multidimensional expansion of a phenylene‐like scaffold.

**Scheme 1:**
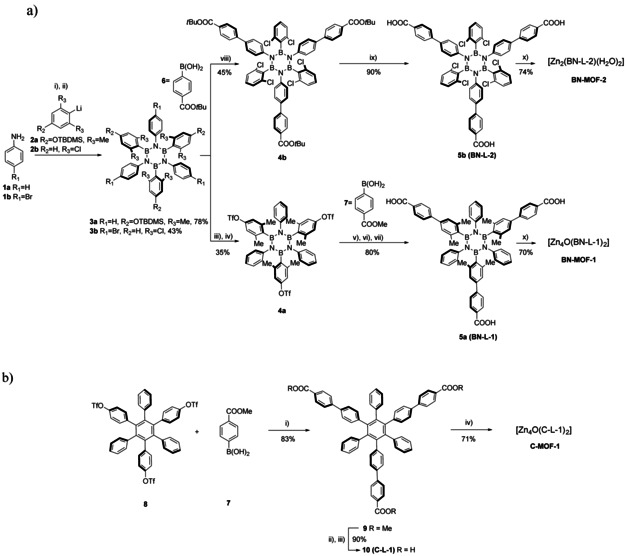
a) Synthesis of three‐carboxyl borazine linkers **BN‐l‐1** (**5 a**) and **BN‐L‐2** (**5 b**), and corresponding **BN‐MOF‐1** and **BN‐MOF‐2**: i) BCl_3_, toluene, reflux, 18 h; ii) **2**, THF, −84 °C to r.t., 24 h; iii) **3 a**, TBAF, THF, r.t., 2 h; iv) Tf_2_O, pyridine, −10 °C, 18 h; v) **7**, [Pd(PPh_3_)_4_], K_2_CO_3_, dioxane, H_2_O, reflux, 48 h; vi) NaOH (1 m), MeOH, THF, r.t., 18 h; vii) HCl (1 m), r.t., 5 min.; viii) **3 b**, [Pd(dba)_2_], K_3_PO_4_, XPhos, THF, 75 °C, 48 h; ix) TFA, CH_2_Cl_2_, r.t., 18 h; x) Zn(NO_3_)_2_⋅6 H_2_O, DMF/NMP, 85 °C, 72 h. b) Synthesis of three‐carboxyl full carbon linker **C‐L‐1** (**10**) and **C‐MOF‐1**. i) [Pd(OAc)_2_], K_3_PO_4_, XPhos, THF, 75 °C, 18 h; ii) NaOH (1 m) MeOH, THF, r.t., 16 h; iii) HCl (1 m), r.t., 5 min; iv) Zn(NO_3_)_2_⋅6 H_2_O, DMF/NMP, 85 °C, 72 h.

With the idea of developing BN‐doped porous materials with large surface areas, we conjectured that aromatic ligands developing in two dimensions and laterally exposing functional groups could allow the formation of MOFs with large surface areas (Figure 1). Building on previous works[^8a^, ^21^] describing moisture‐stable borazine‐based oligophenylenes decorated with *ortho*‐methyl substituents at the inner B‐aryl rings (Figure 1), we conjectured that also borazines could be used as suitable ligands to engineer BN‐doped MOFs if properly equipped with coordinating moieties. Considering that carboxylic acid moieties are well‐known to form persistent Zn_4_O(COO)_6_ metal clusters in MOFs with high porosity,[^19^, ^22^] we designed borazine tricarboxylic acid ligands bearing *ortho*‐methyl and *ortho*‐chloride protecting groups in the B‐aryl rings (Scheme 1). When *ortho* substituents were present, the coordinating carboxylic groups were placed on the *para* positions of extra aryl groups at the outer shell, that are bonded to the B‐ and *N*‐biaryl groups to form **BN‐L‐1** and **BN‐L‐2** linkers, respectively (Scheme 1). As *ortho*‐unsubstituted hexaphenyl borazines cannot be used to prepare MOFs due to their chemical sensitivity toward hydrolysis, we envisaged to prepare an isostructural full‐carbon analogue (**C‐L‐1**) as a reference, in which the aryl rings in the inner shell of the ligand do not bear any *ortho* substituents (i.e., methyl or chlorine groups). When **BN‐L‐1** and **C‐L‐1** linkers are mixed with Zn(NO_3_)_2_⋅6 H_2_O salt, two three‐dimensional porous MOFs **BN‐MOF‐1** and **C‐MOF‐1** with differently interpenetrated structures were obtained. Interestingly, linker **BN‐L‐2** gave a 2D porous MOF (**BN‐MOF‐2**), in which the ligands are held together through Zn_2_(CO_2_)_3_ clusters.

### Synthesis

The borazine precursors of the newly designed trigonal carboxylic linkers used for the synthesis of the reported BN‐MOFs were prepared by following a modified procedure^[21c]^ derived from the original work by Groszos and Stafiej.^[23]^ It was anticipated that these borazine precursors bearing either aryl halide or aryl triflate substituents at the N or B atoms would lead to the targeted organic linkers by performing a Suzuki cross‐coupling reaction with suitable organoboron derivatives.^[21c]^ Thus, targeted borazine tricarboxyl linker **BN‐L‐1** (**5 a**) was obtained in 80 % yield by Suzuki cross‐coupling between molecule **4 a** and 4‐methoxycarbonyl‐phenylboronic acid **7** followed by hydrolysis of the methyl esters. Tris(triflate)‐substituted borazine **4 a** was prepared in 35 % yield^[21e]^ from (TBDMS)‐protected borazine **3 a** through the removal of the TBDMS protecting group with TBAF and subsequent esterification reaction with Tf_2_O in pyridine. Precursor (TBDMS)‐protected borazine **3 a** was obtained in 78 % yield by reacting TBDMS‐protected 2,6‐dimethylphenyl lithium derivative **2 a** with the triphenyl trichloroborazine intermediate that had been formed from the reaction of aniline **1 a** with BCl_3_ in refluxing toluene (Scheme 1 a).

Similarly, tricarboxyl hexachloro‐borazine **BN‐L‐2** (**5 b**) was synthesised by Suzuki cross‐coupling between tris(bromo)‐substituted hexachloro‐borazine **3 b** and 4‐tert‐butoxycarbonyl‐phenylboronic acid **6**, followed by hydrolysis of the obtained tris(*t*Bu‐ester) substituted borazine **4 b** in the presence of TFA, resulting in a 40 % yield over two steps (Scheme 1 a). The synthesis of the precursor tris(bromo)‐substituted hexachloro‐borazine **3 b** was achieved in 43 % yield by reacting the tris(bromo)‐substituted trichloroborazine intermediate, formed by reaction of 4‐bromoaniline **1 b** with BCl_3_ in refluxing toluene, and organolithium derivative **2 b** obtained by lithium‐halogen exchange reaction from bromo‐2,6‐dichlorobenzene using *t*BuLi. Single crystals suitable for XRD analysis could be obtained for linker **BN‐L‐1** by slow diffusion of H_2_O into a DMSO solution. The molecular structure could be determined, confirming the trigonal rigid shape of the borazine ligand (Figure S18). Isostructural full‐carbon analogue linker **C‐L‐1** was also prepared in 75 % yield by Suzuki cross‐coupling reaction between hexaphenylbenzene triflate derivative **8** ^[21d]^ and 4‐methoxycarbonyl‐phenylboronic acid **7**, followed by hydrolysis of the methyl esters groups (Scheme 1 b).

### MOFs synthesis and characterisation

**Figure 2:**
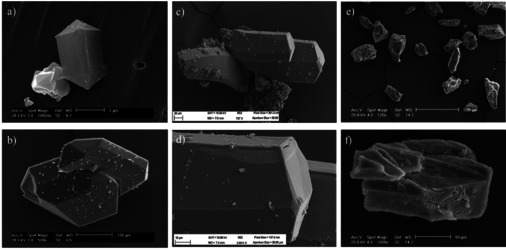
SEM images of the crystals of a,b) **BN‐MOF‐1**; c,d) **BN‐MOF‐2**; e,f) **C‐MOF‐1**.

The MOFs were synthesised by the solvothermal method proposed by O. Yaghi and co‐workers.^[19]^ The reaction conditions were slightly modified^[20]^ and N,N’‐diethylformamide (DEF) was replaced with DMF (Schemes 1 a,b). Considering that the heat transfers that take place during the chemical reaction can affect not only the crystal size, but also the morphology and the phase purity of the porous materials,^[24]^ to ensure the reproducibility the syntheses have been performed in the same sealed vial heated by a bath of non‐flammable silicon‐based oil, keeping the same amount, ratio and concentration of the starting materials. In a typical procedure, the organic linker (respectively **BN‐L‐1**, **BN‐L‐2**, **C‐L‐1**) was dissolved in a mixture 1:1 DMF/NMP, then Zn(NO_3_)_2_⋅6 H_2_O was added and the reaction mixture was heated at 85 °C. After 72 h, colourless crystals were obtained. Porous materials **BN‐MOF‐1**, **BN‐MOF‐2** and **C‐MOF‐1** were isolated in 70 %, 74 % and 71 % yield, respectively. The obtained crystals were firstly analysed by optical (Figure S16) and scanning electron microscope (SEM). The SEM images of **BN‐MOF‐1**, **BN‐MOF‐2** and **C‐MOF‐1** (Figures 2 a‐b, c‐d, and e‐f, respectively) feature their crystalline shape with maximum dimension between 0.5 and 0.6 mm. While both **BN‐MOFs** show more regular prismatic shapes, **C‐MOF‐1** displays more irregular edges.

**Figure 3:**
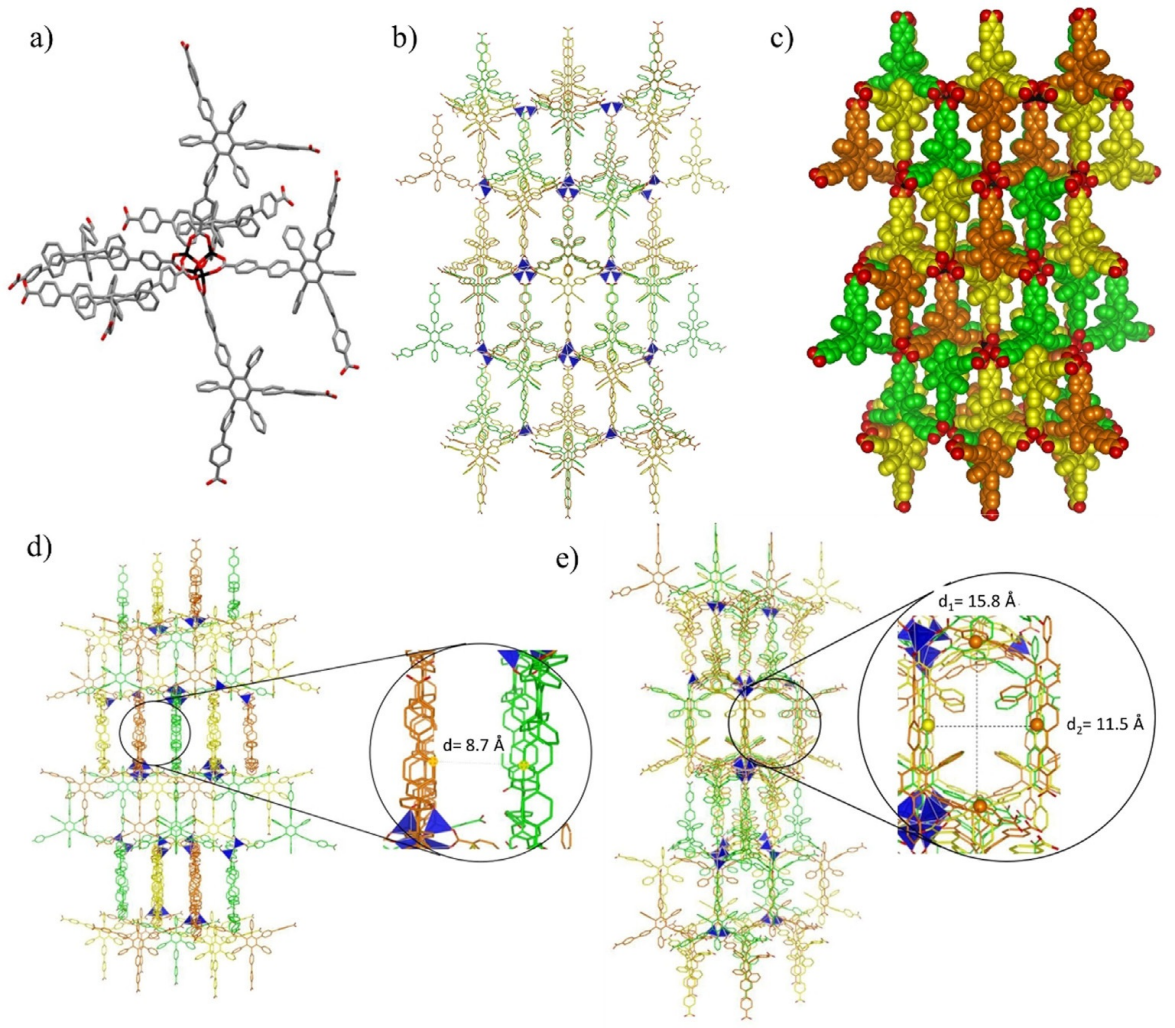
Crystal structure of **C‐MOF‐1** a) Capped sticks representation of a fragment of crystal structure of **C‐MOF‐1** representing the octahedral arrangement of the **C‐L‐1** linkers around the Zn cluster. Space group: *Fdd2*. b) Capped sticks and c) space‐fill representations of the packing view along the *a* axis showing three interpenetrating networks. d) View of the packing showing the distance between the networks and e) view along the *ab* face diagonal showing the cross section of the channels. The distances between the highlighted carbon atoms have been determined from the crystal structure taking into consideration the vdW radii. Colour code: C grey or coloured in orange, green and yellow for the two different networks; pink: B, blue: N, red: O, black or dark blue tetrahedron: Zn. H atoms were omitted for clarity.

The crystals structure for all three MOFs could be determined by single crystal XRD analysis using synchrotron radiation (see Supporting Information). **C‐MOF‐1** formed crystals with an orthorhombic lattice belonging to the *Fdd2* space group showing *ant* topology. A fragment of the structure, depicted in Figure 3 a, shows how six organic linkers are arranged around the distorted octahedral Zn_4_O(COO)_6_ cluster with each bi‐dentate carboxyl moiety chelating two Zn^II^ ions in a bridging fashion. **C‐MOF‐1** shows an interpenetrated structure, where three networks composed by three infinite structurally regular motifs (highlighted in orange, yellow and green, Figure 3 b,c) are inextricably entangled. The distance between each interpenetrated network is approximately 8.7 Å (Figure 3 d), with each network seemingly interacting through vdW interactions. The arrangement of the interpenetrating networks reveals an open porous structure as shown in Figure 3. Theoretical calculations performed with the Platon^[25]^ software indicate that the solvent accessible volume accounts for the 63 % (26 325 Å^3^) of the unit cell volume (41 771 Å^3^). Thus, the framework atoms in **C‐MOF‐1** occupy only the 37 % of the available space in the crystal lattice, with a calculated density of 0.65 g cm^−3^. Indeed, the interpenetration of such entangled networks generates the formation of channels with cross section dimensions of approximately 11.5×15.8 Å that run along the direction of the *ab* face diagonal (110 Miller plane) (Figure 3 e).

**Figure 4:**
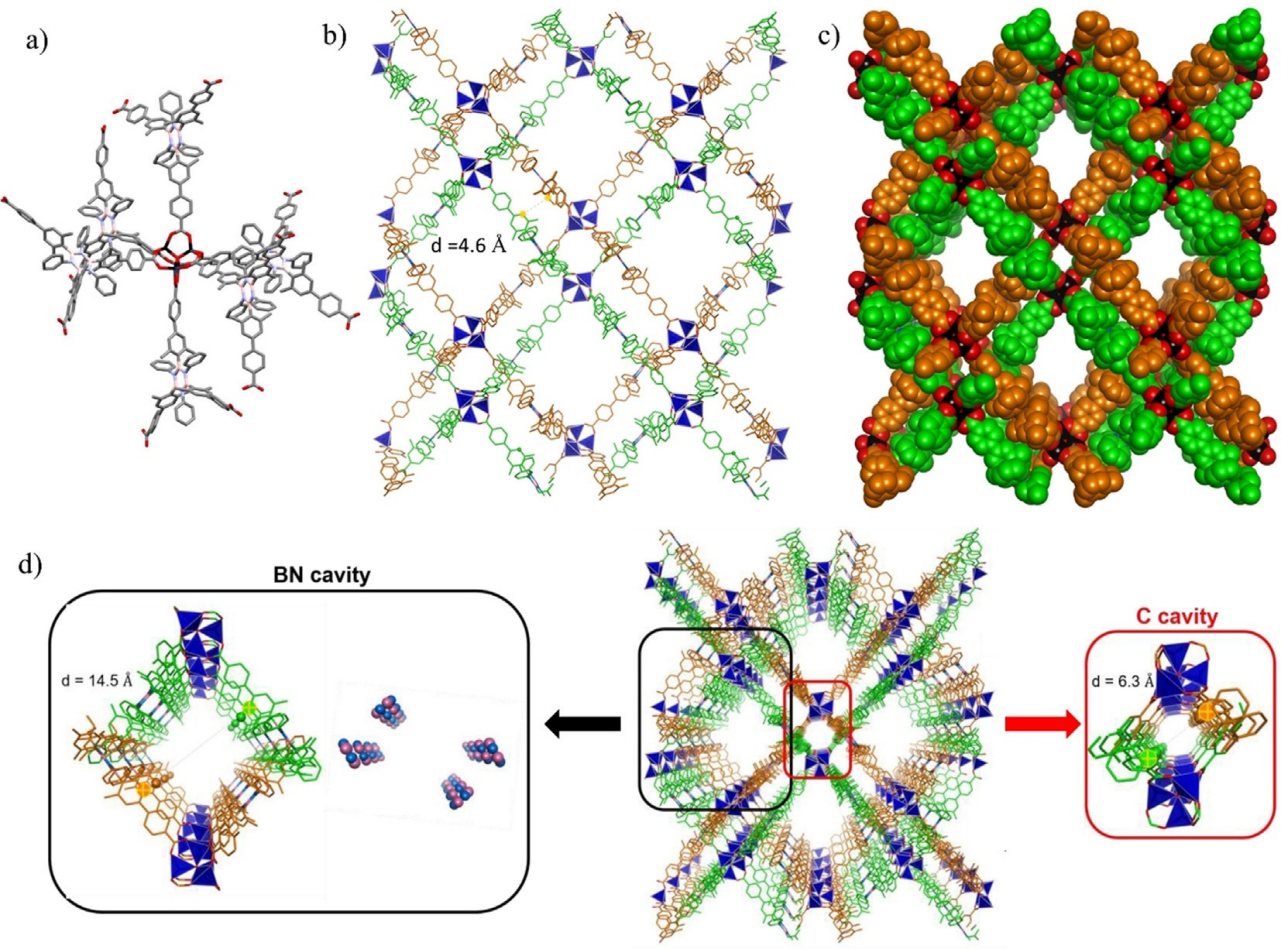
Crystal structure of **BN‐MOF‐1**. a) Capped sticks representation of a fragment of **BN‐MOF‐1** crystal structure showing the octahedral arrangement of **BN‐L‐1** linkers around the Zn cluster. Space group: *Pnnm*. b) Capped sticks and c) space‐fill representation of the two interpenetrating networks d) highlight of the C‐ and BN‐cavities within the structure, the distances between the carbon atoms highlighted have been determined from the crystal structure taking into consideration the vdW radii. The crystal structure is represented in capped sticks. Colour code: C grey or coloured in orange and green for the two different networks; pink: B, blue: N, red: O, black or dark blue tetrahedron: Zn. H atoms were omitted for clarity.

**BN‐MOF‐1**, instead, formed crystals with orthorhombic symmetry analogously to **C‐MOF‐1** but belonging to the *Pnnm* space group and with *rtl* topology. As observed for **C‐MOF‐1**, also for **BN‐MOF‐1**, six organic linkers are arranged around a slightly distorted octahedral Zn_4_O(COO)_6_ cluster, with each bi‐dentate carboxyl moiety bridging two Zn^II^ ions (Figure 4 a). Interestingly, these octahedral fragments extend to form two identical doubly interpenetrating three‐dimensional networks, composed by two infinite structurally regular motifs (highlighted in orange and green, Figures 4 b,c) inextricably entangled. The extended three‐dimensional interpenetrating networks are entangled in such a way that the crystals exhibit a remarkably open three‐dimensional porous structure (Figure 4). Calculations with the Platon software revealed that the solvent accessible void volume accounts for 68 % (19 182 Å^3^) of the total unit cell volume (28 344 Å^3^), when a probe with 1.2 Å radius is used and vdW radii for all atoms (2.25, 1.20, 1.70, 1.52, 1.55, and 1.63 Å for Zn, H, C, O, N and B atoms, respectively) are taken into consideration.

Hence, the framework atoms in **BN‐MOF‐1** occupy only a small fraction (32 %) of the available space in the crystal, with a calculated density of 0.52 g cm^−3^. The non‐framework space is divided into two different types of channels, namely C‐decorated cavities, and BN‐decorated cavities, that run along the crystal *c*‐axis (Figure 4 d). Metal clusters belonging to the two networks and phenylenes moieties form the walls surrounding the C‐cavities (highlighted in red, Figure 4 d), which have a pore diameter of approximately 6.3 Å. The BN‐decorated cavities are larger than those of the C‐counterpart, featuring a diameter of approximately 14.5 Å (highlighted in black, Figure 4 d). The borazine rings are arranged along the walls of the channels exposing the B_3_N_3_ surface towards the centre of the pores. The presence of the *ortho*‐methyl substituents on the B‐aryl substituents reduces the conformational degree of freedom about the C−B bonds. Whereas the dihedral angles between the borazine plane and the inner aryl substituents vary from 75° to 90° in **BN‐MOF‐1**, **C‐MOF‐1** displays dihedral angles ranging from 64° to 90° between the central benzene core and the lateral aryl substituents. In both **BN‐MOF‐1** and **C‐MOF‐1** MOFs, the interweaving might induce strengthening of the architecture as reported for MOFs formed by large linkers,^[26]^ even though only vdW interactions between the two frameworks, which are displaced from one another with a minimum distance of approximately 4.6 Å (Figure 4 b), are present.^[27]^

**Figure 5:**
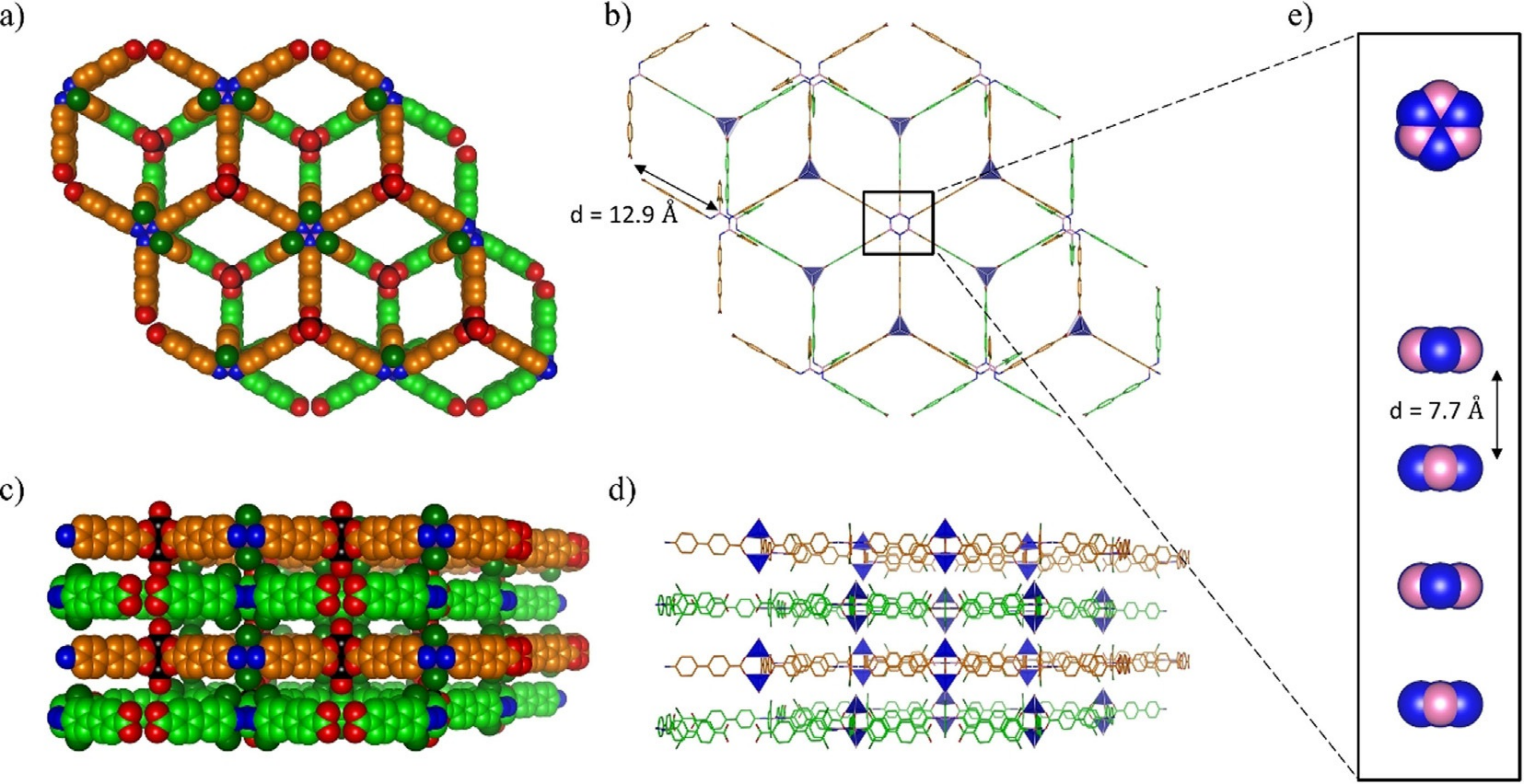
a) Space‐fill and b) capped sticks representation of the top view of **BN‐MOF‐2** crystal structure showing the hexagonal networks and the rhombic channels resulting from the relative 60° rotation of borazine cores in each layer; c) space‐fill and d) capped sticks representation of the side view of **BN‐MOF‐2** showing the layered structure; e) zoomed inset of the borazine cores highlighting the interlayer distance; colour code: C grey or coloured in orange and green for the two different networks; pink: B, blue: N, red: O, black or dark blue tetrahedron: Zn. H atoms were omitted for clarity.

Surprisingly, **BN‐MOF‐2** presents a completely different structure with a trigonal lattice belonging to the *P* $\bar{3}$ *m*1 space group. The threefold ligand symmetry perfectly matches the crystallographic ternary axis of the space group. In this case, three borazine linkers are arranged adopting a trigonal shape around a rare dinuclear Zn_2_(COO)_3_(H_2_O)_2_ secondary building unit,^[28]^ with each bi‐dentate carboxyl moiety bridging two zinc ions and with one water molecule completing each Zn coordination sphere. The networks extend in two dimensions forming a layered structure with a (6,3) topological honeycomb (*hcb*) net based on Zn_2_(COO)_3_(H_2_O)_2_ cluster as a node (Figures 5 a‐b), which has already been reported for the analogous tripodal linker 4,4’,4’’‐(benzene‐1,3,5‐triyl‐tris(benzene‐4,1‐diyl))‐tribenzoic acid.^[29]^ In the **BN‐MOF‐2** crystals the ligands on different layers, which are 7.7 Å apart, are stacked in eclipsed mode along the crystallographic *c* axis (Figure 5 c–e). Adjacent borazine cores are rotated by 60° to minimize steric repulsions between sidechains and to maximize the halogen contacts as previously reported for chlorinated aromatics.^[30]^ This arrangement generates a 3D open porous structure with resulting rhombic channels of approximately 12.9 Å side dimension running along the c‐axis of the crystals (Figures 5 a,b). Calculations with the Platon software revealed that the solvent accessible void volume accounts for 68 % (5236 Å^3^) of the total unit cell volume (7700 Å^3^).

The thermal stability of the three MOFs was evaluated by thermal gravimetric analysis (TGA). All of them MOFs show remarkably high thermal stability, with frameworks decomposition happening at temperatures above 400 °C under N_2_ atmosphere. The TGA trace for **BN‐MOF‐1** clearly shows two distinct weight loss steps (Figure S26 a). The first change, corresponding to 16 % of weight loss, occurred between 50 and 150 °C and can be attributed to the gradual release of the included DMF and NMP solvent molecules. The second step, corresponding to a weight loss of 58 %, was observed at approximately 430 °C and can be attributed to the decomposition of the organic linkers. For **BN‐MOF‐2** gradual elimination of the inclusion solvent, corresponding to 12 % weight loss, was observed between 50 and 250 °C and decomposition of the framework at approximately 450 °C (Figure S26 b). The TGA trace for **C‐MOF‐1** exhibits two steps in the temperature range between 20 °C and 220 °C that correspond to 5 % and 6 % weight loss, which can be attributed to the gradual loss of the inclusion DMF and NMP solvents. (Figure S26 c). A third weigh loss step of 58 % corresponding to the decomposition of the organic framework occurred above 400 °C.

Powder diffraction analysis (PXRD) of freshly prepared samples of **BN‐MOF‐1** and **C‐MOF‐1**, which had been dried under air for 12 h, were also measured to confirm the bulk crystallinity and the stability of the frameworks structures (Figure S27). The experimental data were compared with the simulated powder patterns generated from single‐crystal X‐ray data. Although some similarities in the peaks positions were found for **BN‐MOF‐1**, a mismatch between the relative intensities and a general broadening of the peaks could also be observed (Figure S27). This could be due to partial amorphisation of the material.^[31]^ However, one cannot exclude that differences in peak intensity might arise because the structure is simulated in the absence of guest solvent molecules in the pores, while the experimental pattern is obtained in the presence of solvent molecules, as also confirmed by TGA analysis. Furthermore, additional peaks, which are not found in the simulated pattern, could be observed between 2*θ* 14 and 25° and may indicate a substantial alteration of the **BN‐MOF‐1** structure. As previously reported, Zn_4_O(COO)_6_‐based MOFs demonstrated poor moisture stability, thus suggesting that **BN‐MOF‐1** might also not be very stable under air storage conditions and undergoes partial decomposition due to hydrolysis in the presence of ambient humidity with a consequent partial structural collapse.^[32]^ This is consistent with IR results that show a sharp peak at 3660 cm^−1^ and a broad band at approximately 3400 cm^−1^ that can be attributed to strongly bonded framework water molecules and adsorbed water, respectively (Figure S29). The IR spectrum of an activated sample was also measured and, while it did not show anymore the broad band due to adsorbed water, the peak at 3660 cm^−1^ corresponding to strongly bonded water could still be observed (Figure S30). Moreover, a weak band at 1689 cm^−1^ corresponding to the C=O stretching of the protonated ligand was visible, indicating partial hydrolysis of the framework.[^32a^, ^33^] Similarly, for **C‐MOF‐1** a good agreement between some of the peaks positions in the theoretical and the experimental PXRD patterns was found, namely at 2θ of 7.1 and 7.8°, but again broadening of the peaks and a mismatch in their intensity was also observed (Figure S27). As already noted for **BN‐MOF‐1**, these differences could arise from partial structural collapse but also from the presence of solvent molecules in the cavities of the framework, as proved by TGA analysis, for the measured PXRD pattern as oppose to those obtained through simulation. The IR spectrum of a sample of as‐synthesised **C‐MOF‐1** showed a broad band at about 3300 cm^−1^ due to adsorbed water, but not the sharp peak at about 3650 corresponding to strongly bonded water (Figure S34). Likewise, in the IR spectrum of an activated sample of **C‐MOF‐1** a broad band at about 3300 cm^−1^ was still present but, again, not the peak corresponding to strongly bonded water. In addition, a small band at about 1734 cm^−1^ was found (Figure S35)_._ These observations suggest that protonated organic linkers are present, most likely because of the partial hydrolysis of the framework. An activated sample of **BN‐MOF‐2**, which had been evacuated for 2 h at 403 K, was also analysed by PXRD (Figure S27). Only two weak peaks at 2*θ* 8.2 and 9.2°, which did not match with any peaks in the simulated pattern, could be observed suggesting that the 3D arrangement of **BN‐MOF‐2** is not stable upon activation. SEM images of the activated sample show fracturing of the crystals and especially lines along their c‐axis. These are most probably due to the tendency of the layered structure to split along this direction overcoming the weak interlayers forces, leading to an exfoliation of the MOF layers that contributes to the formation of much smaller particles with hexagonal‐like sheets shape (Figure S36).

**Figure 6:**
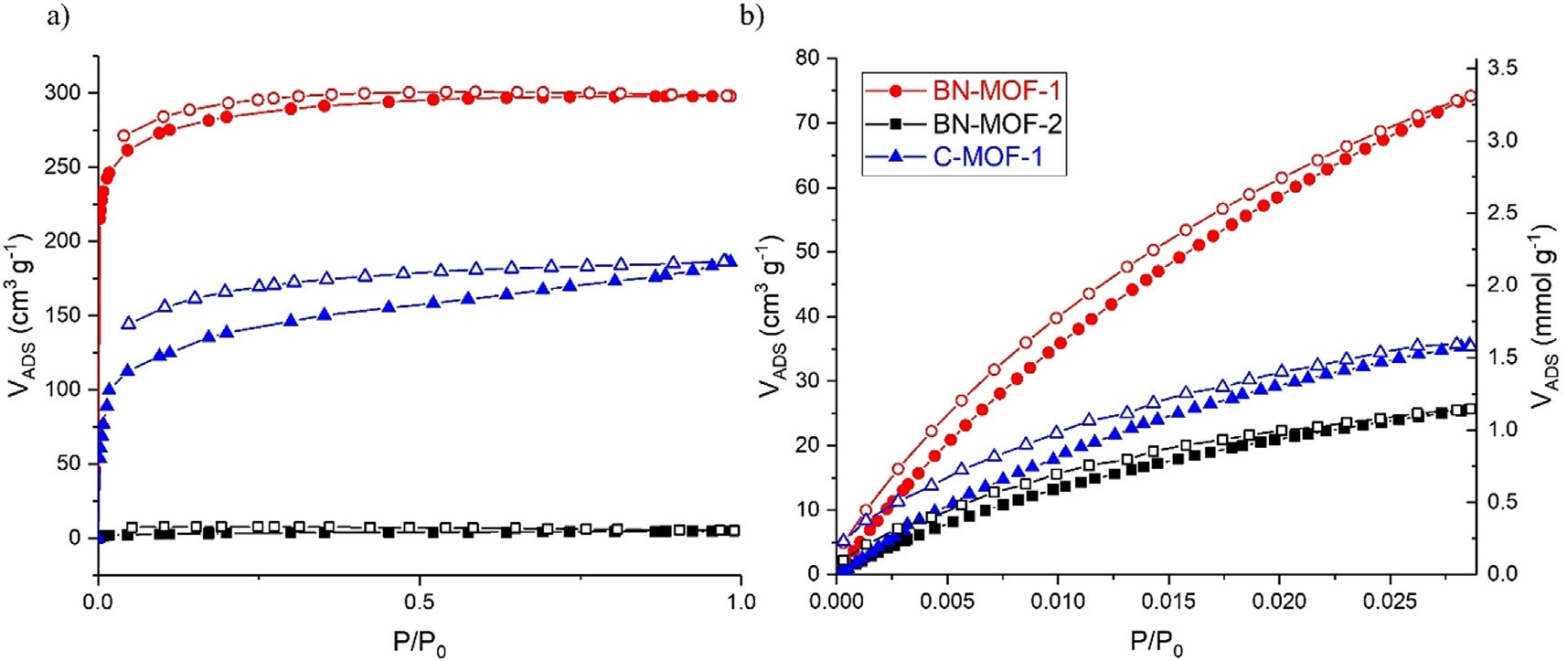
a) N_2_ adsorption isotherms at 77 K and b) CO_2_ adsorption isotherms at 273 K for **BN‐MOF‐1**, **BN‐MOF‐2**, and **C‐MOF‐1**.

Nitrogen adsorption isotherms on activated samples of **BN‐MOF‐1**, **BN‐MOF‐2**, **C‐MOF‐1** were measured to assess their structural stability and permanent microporosity. Although networks interpenetration is considered detrimental to high porosity,^[34]^ the low crystal density and large void volumes calculated from XRD analysis still provided a good indication of potential large surface areas for these materials. Additionally, calculations of the geometric accessible surface areas with the Poreblazer_v3.0.2 program,^[35]^ using a N_2_ size probe molecule with 3.72 Å vdW diameter, provided results that supported these anticipations (Table 1). Before gas adsorption measurements, the samples were activated by exchanging the included DMF/NMP solvents with acetone and then evacuated under high vacuum, before at r.t. and then at 403 K. **BN‐MOF‐1** and **C‐MOF‐1** exhibited a type Ia N_2_ adsorption isotherm,^[36]^ with a sharp gas uptake at very low partial pressure (*P*/*P*_0_<0.05) (Figure 6 a). While no significant hysteresis upon desorption of gas from the pores was observed for **BN‐MOF‐1**, **C‐MOF‐1** showed a relatively pronounced hysteresis, which might be indicative of some deformation of the reticulate during the adsorption step. **C‐MOF‐1** showed a relatively slow kinetic of adsorption in comparison with **BN‐MOF‐1**. This feature along with the pronounced hysteresis suggests that some pores might not be readily accessible. The apparent Brunauer–Emmett–Teller (BET) surface areas were calculated as 1096 and 509 m^2^ g^−1^, and a total pore volume of 4.831×10^−1^ and 2.887×10^−1^ cm^3^ g^−1^, for **BN‐MOF‐1** and **C‐MOF‐1**, respectively. Whilst it was expected for **C‐MOF‐1** having a lower surface area than that of **BN‐MOF‐1**, higher values were anticipated for both on the base of the obtained geometric values calculated from the crystal structure (Table 1). These discrepancies seem to suggest that a partial structural collapse might be happening either before, as indicated by PXRD analysis, or/and after samples activation. **BN‐MOF‐2** showed a very uneven adsorption isotherm with extremely low N_2_ uptake (5.25 cm^3^ g^−1^) and a calculated apparent BET surface area of 12 m^2^ g^−1^ (Figure 6 a). The total pore volume at *P*/*P*_0_=0.98 was calculated to be of 7.82×10^−3^ cm^3^ g^−1^. The very low surface area, which greatly differs from the calculated geometric one, might be attributed to structural collapse (see also PXRD and SEM analysis).

**Table 1:** Porosity data for **BN‐MOF‐1**, **BN‐MOF‐2**, and **C‐MOF‐1**.

Compound	Void volume^[a]^ [%]	Crystal density^[a^ [g cm^−3^]	*SA*_geo_^[b]^ [m^2^ g^−1^]	*SA*_BET_ [m^2^ g^−1^]	*V*_p_^[c]^ [cm^3^ g^−1^]
**BN‐MOF‐1**	68	0.52	3051	1096	0.48
**BN‐MOF‐2**	68	0.55	3509	12 (270^[d]^)	0.008 (0.044^[d]^)
**C‐MOF‐1**	63	0.65	2746	509	0.29

[a] Calculated with Platon with 1.2 Å radius probe. [b] Accessible geometric surface area calculated with Poreblazer_v3.0.2 with a nitrogen molecule size probe (diameter 3.72 Å), [c] *V*p is the measured pore volume, [d] BET surface area and pore volume at *P*/*P*_0_=0.028 calculated from CO_2_ isotherm.

Even though these MOFs exhibit moderate porosities when compared with ultrahigh porous MOFs constructed from tripodal organic linkers such as MOF‐177^[19]^ and MOF‐200,^[20]^ it was of interest to study the CO_2_ uptake. Thus, CO_2_ adsorption isotherms at 273 and 298 K up to 1 bar were measured for all three MOFs (Figure 6 b). **BN‐MOF‐1** shows a CO_2_ uptake of 3.31 and 1.89 mmol g^−1^ at 273 and 298 K, respectively, that is higher than those observed for MOF‐5 (i.e. 0.82 mmol g^−1^) and MOF‐177 (i.e. 1.7 mmol g^−1^)^[37]^ under similar experimental conditions.

The curve did not show an important hysteresis, that is, the adsorption and desorption curves follow almost the same path, which suggests that the adsorption process is essentially reversible. **C‐MOF‐1** exhibited halved CO_2_ uptakes, 1.60 and 0.95 mmol g^−1^ at 273 K and 298 K, compared to **BN‐MOF‐1**. At last, **BN‐MOF‐2** showed higher CO_2_ adsorption capacity than that for N_2_, with uptakes of 1.15 and 0.80 mmol g^−1^ at 273 and 298 K respectively_._ It is possible that the higher measurement temperature (273 K for CO_2_ vs. 77 K for N_2_) produced some expansion in the material that permits a higher adsorption for CO_2_, which has a smaller kinetic diameter than N_2_. This is somewhat confirmed by the more pronounced hysteresis of the CO_2_ adsorption at 298 K, which might be due to the swelling of the material.^[38]^ Although rarely done, the BET surface calculation from the CO_2_ adsorption curve is considered reliable,^[39]^ therefore a BET surface area of 270 m^2^ g^−1^ with a total pore volume at *P*/*P*_0_=0.028 of 4.36×10^−2^ cm^3^ g^−1^, could be calculated from the CO_2_ isotherm at 273 K. For all three MOFs, it could be observed that the adsorption isotherm does not reach a plateau in the pressure range investigated, indicating that they can adsorb more CO_2_ at higher partial pressures. In addition, the pore size distribution (PSD) was calculated from the adsorption isotherms at 273 K via nonlocal density functional theory (NLDFT) for all three MOFs (Figure S37). For **BN‐MOF‐1**, the distribution shows two well defined peaks centred at 5.7 and 8.6 Å, that would suggest the presence of a regular structure with defined and accessible channels. For **C‐MOF‐1**, one well‐defined peak centred at 5.7 Å almost overlapped with a smaller one centred at 6.3 Å and a third broad one centred at 8.2 Å could be observed. This pattern suggests the presence of intricated channels in the structure, which seems to correlate well with the slow N_2_ adsorption kinetic and to the three networks concatenated structure. At last, for **BN‐MOF‐2** the distribution showed a series of peaks in the 4.8–7 Å range and a third broad one centred at 8.2 Å. The low fraction of small pores seems to confirm that the material is little porous, with an irregular structure as suggested by a scattered distribution of peaks. Finally, to evaluate whether the polarity of the BN nitrogen bonds in the borazine cores within the MOFs was inducing an increased affinity towards CO_2_, isosteric heats of adsorption from the CO_2_ isotherms at 273 K and 298 K were calculated. It has been found that the heats of adsorption are approximately 28 KJ mol^−1^ for **BN‐MOF‐1** and 32 KJ mol^−1^ for **BN‐MOF‐2** and **C‐MOF‐1**, thus similar to that of MOF‐5 (34 KJ mol^−1^).^[40]^ These values seem to indicate that the polar BN bonds are not substantially strengthening the interactions with CO_2_ in **BN‐MOF‐1** as compared to **C‐MOF‐1**. This aligns with the idea that the BN couples bonds are shielded by the Me groups, thus of difficult accessibility for CO_2_ physisorption.

## Conclusions

In summary, we have described the first example of the use of borazine unit to prepare crystalline porous MOFs from specifically designed borazine carboxylate linkers. Moreover, to evaluate the influence of the borazine rings on the gas adsorption properties, the analogous full‐carbon carboxylate linker and the corresponding MOF have also been prepared. The structure of the three MOFs was unambiguously determined by single crystal X‐ray analysis. **BN‐MOF‐1** showed a 3D open porous framework, resulting from the double interpenetration of two identical networks with consequent formation of BN‐cavities with the borazine moieties arranged along the walls of the channels. On the other hand, **BN‐MOF‐2** exhibited a crystal packing where 2D metal organic networks are arranged to form large rhombic channel running perpendicular to the networks layers. Finally, **C‐MOF‐1** crystals showed a structure where three networks composed by three infinite structurally regular motifs are inextricably entangled. **BN‐MOF‐1**, **C‐MOF‐1** and **BN‐MOF‐2** exhibited BET surface areas of 1096, 509 and 12 m^2^ g^−1^ and CO_2_ adsorption at 273 K of 3.31, 1.60 and 1.15 mmol g^−1^, respectively. The CO_2_ uptake by **BN‐MOF‐2** is considerably high considering the extremely low BET surface area, indicating a stronger affinity of this material for CO_2_. The experimental BET surfaces areas are lower than those calculated geometrically, revealing some possible network instability following post synthetic treatment and activation, as also indicated by powder diffraction and IR studies. These observations suggest that different activation processes might help in getting higher porosity and surface areas. Taken together, these results suggest that the borazine core can be used as versatile scaffold to build ligands for 2D and 3D BN‐doped MOFs and since it can be structurally modified by organic functionalisation, it might constitute a seed for preparing porous BN materials.

## Experimental Section

**Crystallographic data**: Deposition numbers 2038664, 1418729, 2011413, 2011411, and 2011414 contain the supplementary crystallographic data for this paper. These data are provided free of charge by the joint Cambridge Crystallographic Data Centre and Fachinformationszentrum Karlsruhe Access Structures service.

## Conflict of interest

The authors declare no conflict of interest.

## Supporting information

As a service to our authors and readers, this journal provides supporting information supplied by the authors. Such materials are peer reviewed and may be re‐organized for online delivery, but are not copy‐edited or typeset. Technical support issues arising from supporting information (other than missing files) should be addressed to the authors.

Supplementary
