# Supplementary material for: BN‐Doped Metal–Organic Frameworks: Tailoring 2D and 3D Porous Architectures through Molecular Editing of Borazines
Source: Chemistry. 2021 Jan 31;27(12):4124–33. doi: 10.1002/chem.202004640 (PMC7986190; doi:10.1002/chem.202004640)
Supplement: Supplementary file 1 — Supplementary [file CHEM-27-4124-s001.pdf]

Chemistry–A European Journal

Supporting Information

BN-Doped Metal–Organic Frameworks: Tailoring 2D and 3D Porous Architectures through Molecular Editing of Borazines

Francesco Fasano,[a] Jacopo Dosso,[a] C. Grazia Bezzu,[a] Mariolino Carta,[b] François Kerff,[a] Nicola Demitri,[c] Bao-Lian Su,[d] and Davide Bonifazi*[a, e]

Table of contents

S1 General remarks 3
S1.1 Material and general methods 3
S1.2 Instrumentation 3
S2 Synthetic procedures and spectral data 4
S2.1 Synthesis of N,N',N''-triphenyl-B,B',B''-tri(2,6-dimethyl-4-(tert-butyl dimethylsilyloxy)phenyl)borazine 3a and N,N',N''-triphenyl-B,B',B''-tri(2,6-dimethyl-4-trifluoromethanesulphonate)phenyl)borazine 4a 4
S2.3 Synthesis of [$Zn_4O(BNTPC)_6$] BN-MOF-1 5
S2.6 Synthesis of N,N',N''-tri(4-(4-carboxyphenyl)phenyl)-B,B',B''-tri(2,6-dichloro)phenylborazine BN-L-2 (5b) 7
S2.7 Synthesis of [$Zn_4O(BNClTPC)_6$] BN-MOF-2 8
S2.8 Synthesis of 1,3,5-tris-(4-(4-metoxycarbonyl-phenyl)phenyl)-2,4,6-phenylbenzene 9.. 9
S2.9 Synthesis of 1,3,5-tris-(4-(4-carboxylphenyl)phenyl)-2,4,6-phenylbenzene C-L-1 (10)10
S2.10 Synthesis of [$Zn_4O(CTCP)_6$] C-MOF-1...................................... 10
S3 Characterization..11
S3.1 Characterization of BN-L-1 11
S3.2 Characterization of 3b...................................... 13
S3.3 Characterization of 4b...................................... 14
S3.4 Characterization of BN-L-2 16
S3.5 Characterization of 9 17
S3.6 Characterization of C-L-1 18
S4 Optical Microscope images..19
S5 Crystallographic data 20
S6 Thermal Gravimetric analysis 28
S7 Powder X-Ray Diffraction..29
S8 Linkers and MOFs Infrared spectra 30
S9 Scanning electron microscope images of activated BN-MOF-2..34
S10 Pores size distribution..34
S11 References 35

S1 General remarks

S1.1 Material and general methods

Chemicals were purchased from *Sigma-Aldrich*, *Acros Organics*, *Fluorochem, TCI*, *Carbosynth* and *ABCR,* and were used as received unless specified otherwise. Solvents were purchased from *Sigma-Aldrich* and *Acros Organics*. Deuterated solvents were purchased from *Eurisotop*. Anhydrous solvents were obtained with a Braun MB SPS-800 solvent purification system and further dried over activated 4 Å molecular sieves. Low temperature baths were prepared using different solvent mixtures depending on the desired temperature: -84 °C with EtOAc/liquid N_2, -78 ºC Et_2O/N_2 liquid, and 0 °C with ice/H_2O. Anhydrous conditions were achieved by drying *Schlenk* lines, 2-neck flasks or 3-neck flasks by heating with a heat gun under vacuum and then purging with nitrogen. The inert atmosphere was maintained using nitrogen-filled balloons equipped with a syringe and needle that was used to penetrate the silicon stoppers used to close the flask necks. The addition of liquid reagents was done by means of dried plastic syringes or by cannulation. Molecular sieves (4 Å) were activated by heating at 165 °C under vacuum (less than 1 mbar) overnight. All reactions were performed in dry conditions and under inert atmosphere unless otherwise stated.

S1.2 Instrumentation

Thin layer chromatography (TLC) was conducted on pre-coated aluminium sheets with 0.20 mm Merk Millipore Silica gel 60 with fluorescent indicator F254.

Column chromatography was carried out using *Merck Gerduran silica gel* 60 (particle size 40-63μm).

Melting points (MP) were measured on a *Büchi Melting Point B-545* or were measured on a *Gallenkamp* apparatus. All of the melting points have been measured in open capillary tubes.

Nuclear magnetic resonance (NMR) ^{1}H, ^{11}B and ^{13}C spectra were obtained on a 300 MHz NMR (*Bruker Fourier*), 400 MHz NMR *(Jeol JNM EX-400*or *Bruker AVANCE III HD),* and 500 MHz NMR *(Jeol NM EX-500R)*. Chemical shifts were reported in ppm according to tetramethylsilane using the solvent residual signal as an internal reference ($CDCl_3$: δ_H = 7.26 ppm, δ_C = 77.16 ppm, CD_2Cl_2: δ_H = 5.32 ppm, δ_C = 53.84 ppm; DMSO-d_6: δ_H = 2.5 and 3.33 ppm, δ_C = 39.52 ppm; acetone-d_6: δ_H = 2.05 and 2.84 ppm, δ_C = 29.84 and 206.26 ppm). Coupling constants (*J*) were given in Hz and were averaged. Resonance multiplicity was described as s (singlet), d (doublet), t (triplet), m (multiplet), br (broad signal). Carbon and boron spectra were acquired with a complete proton decoupling. All spectra were recorded at 25 °C unless specified otherwise. Signals observed in ^{1}H spectra at 1.27 ppm and 0.8 ppm are traces of grease.

Infrared spectra (IR) were recorded on a *Perkin-Elmer Spectrum II FT-IR System UATR*, mounted with a diamond crystal, on a *BIO-RAD FTS-165 apparatus*, or on a *Shimadzu IR Affinity 1S FTIR spectrometer* in ATR mode with a diamond mono-crystal. Selected absorption bands are reported in wavenumber (cm^{-1}).

ESI and API mass spectrometry. High and low resolution ESI or API mass spectrometry were performed on a Waters LCT HR TOF mass spectrometer in the positive or negative ion mode at *Cardiff University*. The analytes were dissolved in a suitable solvent at a concentration of 1 mg/mL and diluted 200 times in methanol (≈ 5 ng/mL). The diluted solutions (1µL) were delivered to the ESI source by a Dionex Ultimate 3000 RSLC chain used in FIA (Flow Injection Analysis) mode at a flow rate of 200 µL/min with a mixture of CH_3CN/H_2O+0.1% of HCOOH (65/35). ESI conditions were as follows: capillary voltage was set at 4.5 kV; dry nitrogen was used as nebulizing gas at 0.6 bars and as drying gas set at 200 °C and 7.0 L/min. ESI-MS spectra were recorded at 1 Hz in the range of 50-3000 *m/z*. Data were processed using Bruker Data Analysis 4.1 software.

TGA. Thermogravimetric analysis was performed using the device Thermal Analysis TGA Q500. The samples, previously dried in air for 12 hours, were heated to 30 °C and kept at this temperature for 30 min, then heated a constant rate of 5° C min^{-1} from 30 to 800 °C, under N_2 flow.

Powder X-ray diffraction (PXRD). Powder X-ray diffraction patterns were obtained on a PANanalytical X`Pert Pro diffractometer using Cu Kα ray source operating at 40 kV and 40 mA. The signal was recorded for 2θ between 10º and 25º with a step of 0.02º. The XRD patterns were analysed following the JCPDS database, all this analysis was carried out at *Cardiff University*.

Scanning electron microscopy (SEM**).** Scanning electron microscopy images were obtained using a Carl Zeiss EVO 40 operated at 25 kV. All samples were mounted on carbon Leit adhesive discs. Images were collected using backscattered and secondary electron detectors. For EDX (X-ray energy dispersive spectroscopy) analysis, high probe currents (up to 25 nA) were required to allow sufficient generation of X-rays. The data was collected using an Oxford EDX analyser coupled to the EVO 40 SEM, all this analysis was carried out at *Cardiff University*.

N_2 and CO_2 adsorption/desorption isotherms were obtained using a Quantachrome Nova 2200e. The gases were supplied by BOC and used without any further purification (N_2 purity > 99.999, CO_2 purity > 99.99%). The samples were measured twice in two different cells, to minimize the error providing the same results. The BET was calculated at a relative pressure $P/P_0 < 0.05$ and it was elaborated using the standard Quantachrome software. The sample were weighed in a Quantachrome adsorption cell and outgassed under vacuum for 2 hours at 403 K

S2 Synthetic procedures and spectral data

S2.1 Synthesis of N,N',N''-triphenyl-B,B',B''-tri(2,6-dimethyl-4-(tert-butyl dimethylsilyloxy)phenyl)borazine 3a and N,N',N''-triphenyl-B,B',B''-tri(2,6-dimethyl-4-trifluoromethanesulphonate)phenyl)borazine 4a

Molecules **3a** and **4a** were synthesised as we previously reported.[1S]

S2.2 Synthesis of N,N',N''-triphenyl-B,B',B''-tri(2,6-dimethyl-4-(4-(carboxy)phenyl)phenyl)borazine BN-L-1 (5a)

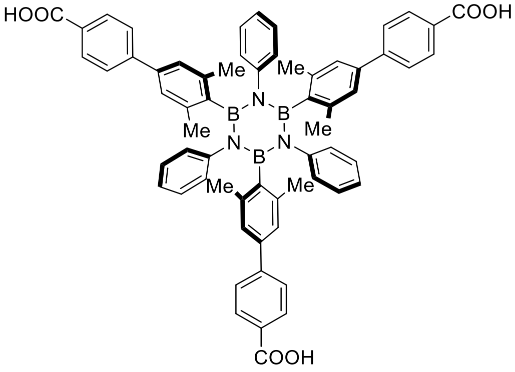

BN-L-1

To a solution of **4a** (0.350 g, 0.33 mmol) in a 5:1 dioxane/H_2O mixture (15 mL), 4-metoxycarbonyl-phenylboronic acid **7** (0.245 g, 1.48 mmol), [Pd(PPh_3)$_4$] (0.038 g, 0.032 mmol) and K_2CO_3 (0.542 g, 3.93 mmol) were added and placed under inert atmosphere of N_2. The reaction mixture was degassed by N_2 bubbling under sonication for 3 h then heated at 105 °C for 48 h. The solution was diluted with EtOAc (50 mL) and washed with H_2O (3 x 100 mL). The H_2O solution was concentrated under reduced pressure. The solid residue was dissolved in a 4:1 mixture of THF/CH_3OH (35mL), then a solution of NaOH (1M) was added dropwise until pH: 13-14 was obtained. The reaction mixture was stirred at room temperature for 18h. Then CH_3OH and THF were removed under reduced pressure and HCl (1M) was added dropwise at 0 °C until pH: 1 was obtained. The white solid was filtered off affording pure **BN-L-1** as a white powder (0.26 g, 80%). MP: > 300 °C. ^{1}H NMR (300 MHz, DMSO) δ: 12.89 (s, 3 H), 7.87 (d, J = 8.3 Hz, 6 H), 7.58 (d, J = 8.3 Hz, 6 H), 7.00-6.98 (m, 12 H), 6.87-6.83 (m, 6 H), 6.74-6.70 (m, 3 H) 2.41 (s, 18 H). ^{13}C NMR (100 MHz, DMSO) δ: 167.1, 145.7, 143.7, 139.5, 138.1, 136.9, 129.7, 129.2, 127.2, 126.3, 126.0, 124.9, 123.5, 23.0. ^{11}B NMR (128 MHz, $CDCl_3$) δ: 30.1. ESI-LRMS: $[M]^+$ calc. for $[C_{63}H_{54}B_3N_3O_6]^+$: 981.43, found: 981.57. FT-IR (solid) ν_{max} (cm^{-1}): 3044, 2911, 1689, 1606, 1357, 1015, 702, 651. Crystals suitable for X-Ray diffraction were obtained from slow evaporation of a H_2O in DMSO solution (See section S5 crystallographic data).

S2.3 Synthesis of [Zn_4O(BNTPC)$_6$] BN-MOF-1

To a microwave vial (Biotage vial, size L x W x H: 1.5 x 1.5 x 8 cm) **BN-L-1** (0.015 g, 0.015 mmol) and $Zn(NO_3)_2 \cdot 6H_2O$ (0.075 g, 0.25 mmol) were added and dissolved in a mixture 1:1 DMF/NMP (4mL). The reaction mixture was heated at 85°C for 72h. The crystals obtained were filtered and dried under air affording [Zn_4O(BNTCP)$_6$] **BN-MOF-1** (0.013 g, 70%). See *section* X-ray crystal data. Solvent exchange

procedure: **BN-MOF-1** was washed thoroughly with a mixture of 1:1 DMF/NMP (30 mL) to remove any non-coordinated three-carboxyl borazine **BN-L-1**. In a centrifuge tube, **BN-MOF-1** and acetone were added and the suspension was left to soak in the new solvent for 20 min. Subsequently, the suspension was washed with centrifugation (5000 rpm, 30 min) and then the supernatant was carefully removed. This procedure was applied for 5 cycles. FT-IR (solid) ν_{max} (cm^{-1}): 3650, 3455, 2983, 1652, 1560, 1350, 1082, 705, 662, 562, 539. BET surface area = 1096 m^2 g^{-1}; total pore volume of 4.831*10^{-1} cm^3 g^{-1}, calculated at P/P_0 = 0. 98288, from N_2 adsorption at 77 K.

S2.4 Synthesis of N,N',N''-tri(4-bromo)phenyl-B,B',B''-tri(2,6-dichloro)phenylborazine 3b

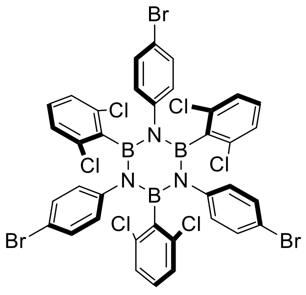

3b

To a solution of 4-bromoaniline **1b** (500 mg, 2.82 mmol) in dry toluene (5.0 mL) under N_2, a solution of BCl_3 (1.0 M solution in heptane; 3.11 mL; 3.11 mmol) was added dropwise at 0 °C. The septum of the Schlenk tube was substituted with a condenser equipped with a $CaCl_2$ trap and the resulting mixture refluxed under N_2 for 18 h. The condenser was then substituted with a septum and the reaction was degassed five times following the freeze-pump-thaw procedure to remove the residual HCl. In parallel, 1-bromo-2,6-dichlorobenzene (631 mg, 2.82 mmol) was dissolved in 8.0 mL of anhydrous THF in a flame-dried Schlenk flask and *t*-BuLi (1.7 M pentane, 3.32 mL, 5.64 mmol) slowly added dropwise at -84 °C. The suspension was stirred for 10 min. at the same temperature. The chloro-borazole intermediate was then cannulated to (2,6-dichlorophenyl)lithium **2b** at -84 °C and allowed to react at this temperature for 30 min, at 0 °C for 30 min and at r.t. for 4 h. The reaction was quenched with water, diluted in CH_2Cl_2, the organic layer washed with H_2O (100 mL × 2) and brine (100 mL). The organic layer was then dried over $MgSO_4$, filtered and evaporated under vacuum. Precipitation of the residue in MeOH afforded pure **3b** as a white solid (397 mg, 43%). MP: > 300 °C. ^1H NMR (300 MHz, $CDCl_3$) δ: 7.27 (d, *J* = 8.5 Hz, 6 H), 7.01 (d, *J* = 8.5 Hz, 6 H), 6.86 (s, 9 H). ^{13}C NMR (75 MHz, $CDCl_3$) δ: 144.0, 136.2, 130.6, 130.5, 128.3, 126.5, 119.5. ^{11}B NMR (128 MHz, CDCl3) δ: 33.7. ASAP$^+$-HRMS: $[M+H]^+$ calc. for $[C_{36}H_{21}B_3N_3Cl_6Br_3]^+$: 980.7635, found: 980.7657. FT-IR (solid) ν_{max} (cm^{-1}): 2999, 2949, 1581, 1552, 1398, 1010, 968, 711, 644, 418, 410. Crystals suitable

for X-Ray diffraction were obtained from slow evaporation of a $CHCl_3$ solution (See section S5 crystallographic data).

S2.5 Synthesis of N,N',N''-tri(4-(4-tert-butoxycarbonylphenyl)phenyl)-B,B',B''-tri(2,6-dichloro)phenylborazine 4b

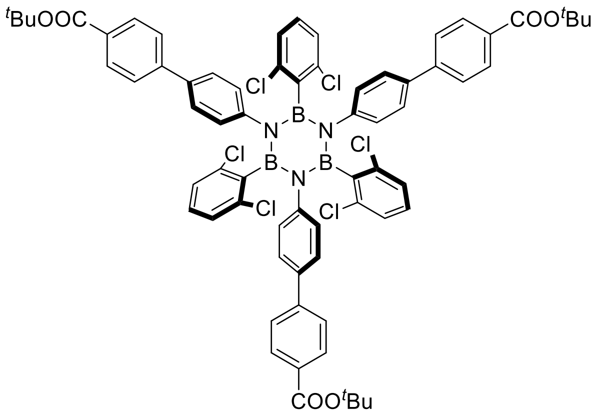

4b

In a Schlenk tube **3b** (300 mg, 0.31 mmol) was added together with (4-(tert-butoxycarbonyl)phenyl)boronic acid (273 mg, 1.23 mmol), K_3PO_4 (262 mg, 1.23 mmol), [Pd(dba)$_2$] (18 mg, 0.03 mmol) and XPhos (29 mg, 0.06 mmol). Anhydrous THF (5 mL) was then added to the solids and the resulting suspension was degassed with N_2 bubbling and sonication for 20 min and then stirred at 75 °C under N_2 for 48 h. The reaction was then diluted with CH_2Cl_2 (50 mL), washed with water (50 mL × 2), brine (50 mL), dried on $MgSO_4$, filtered and the solvents evaporated under reduced pressure. The resulting material was purified via short plug silica gel chromatography (eluent: petroleum ether/CH_2Cl_2 1:1 to CH_2Cl_2/EtOAc 95:5)* to give **4b** as a beige fluffy solid (177 mg, 45 %). MP: >300 °C. ^{1}H NMR (300 MHz, $CDCl_3$) δ: 7.90 (d, J = 8.5 Hz, 6 H), 7.53 (d, J = 8.5 Hz, 6 H), 7.41 (d, J = 8.5 Hz, 6 H), 7.19 (d, J = 8.5 Hz, 6 H), 6.86-6.75 (m, 9 H), 1.58 (s, 27 H). ^{13}C NMR (75 MHz, $CDCl_3$) δ: 165.8, 145.2, 144.5, 136.9, 136.4, 130.4, 130.1, 129.8, 127.2, 126.5, 126.3, 126.1, 81.1, 28.4. The peak of the carbon directly bonded to the boron atom is missing due to the quadrupolar relaxation. ^{11}B NMR (128 MHz, $CDCl_3$) δ: 38.2. AP$^+$-HRMS: [M]$^+$ calc. for $[C_{69}H_{60}{}^{10}B^{11}B_2N_3Cl_6O_6]^+$: 1268.2929, found: 1268.2975. FT-IR (solid) ν_{max} (cm^{-1}): 3047, 2974, 1583, 1552, 1377, 1367, 1024, 771, 732.

*Necessary to add some drops of MeOH to recover all the product from the silica phase.

S2.6 Synthesis of N,N',N''-tri(4-(4-carboxyphenyl)phenyl)-B,B',B''-tri(2,6-dichloro)phenylborazine BN-L-2 (5b)

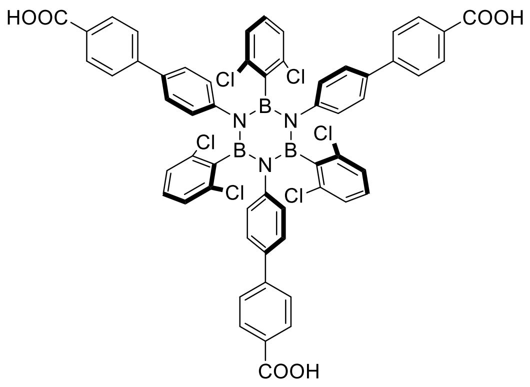

BN-L-2

In a round bottom flask **4b** (100 mg, 0.08 mmol) was added and dissolved in CH_2Cl_2 (5 mL). TFA (0.2 mL, 0.96 mmol) was then added and the reaction stirred at r.t. for 18 h. Compressed air was then used to evaporate 50 % of the solvent, addition of petroleum ether led to the formation of a white precipitate and , filtration of the resulting suspension gave pure **BN-L-2** as a white powder (80 mg, 90 %). MP: >300 °C. ^{1}H NMR (300 MHz, Acetone-*d*6) δ: 7.95 (d, *J* = 8.5 Hz, 6 H), 7.67 (d, *J* = 8.5 Hz, 6 H), 7.51 (d, *J* = 8.5 Hz, 6 H), 7.37 (d, *J* = 8.5 Hz, 6 H), 7.01-6.89 (m, 9 H). ^{13}C NMR (75 MHz, Acetone-*d*6) δ: 167.4, 146.1, 144.9, 137.6, 137.1, 131.6, 131.0, 130.1, 128.1, 127.3, 127.2, 126.8. The peak of the carbon directly bonded to the boron atom is missing due to the quadrupolar relaxation. ASAP^{+}-HRMS: $[M+H]^{+}$ calc. for $[C_{57}H_{37}{}^{10}B^{11}B_2N_3Cl_6O_6]^{+}$: 1101.1129, found: 1101.1077. FT-IR (solid) ν_{max} (cm^{-1}): 3043, 2954, 1685, 1552, 1523, 1375, 1006, 867, 837, 798, 713.66, 651.

S2.7 Synthesis of [$Zn_4O(BNClTPC)_6$] BN-MOF-2

In a microwave vial (Biotage vial, size L × W × H: 1.5 × 1.5 × 8 cm), **BN-L-2** (15 mg, 0.014 mmol) was added with $Zn(NO_3)_2{\cdot}6H_2O$ (69 mg, 0.23 mmol) and dissolved in 4 mL of a 1:1 mixture of DMF/NMP. The vial was sealed, wrapped in aluminium foil and sonicated until complete solubilization of the powders occurred. The vial was then inserted in a pre-heated oil bath at 80 °C and left at the same temperature without stirring for 72 h. The resulting brownish solution containing transparent hexagonal prisms was filtered (paper filter without vacuum) and washed thoroughly with DMF resulting in a transparent crystalline powder of **BN-MOF-2** (13 mg, 74 %). Solvent exchange procedure: the sample was slowly washed with acetone (500 mL) on a paper filter, then dried on air for ~~at least~~ 3 h. FT-IR (solid) ν_{max} (cm^{-1}): 3456, 2932, 2862, 1655, 1649, 1551, 1090,1007, 708, 658, 586, 405. BET surface area = 12 m^{2} g^{-1}; total pore volume of 7.82*10^{-3} cm^{3} g^{-1}, calculated at P/P_0 = 0.9893, from N_2 adsorption at 77 K.

S2.8 Synthesis of 1,3,5-tris-(4-(4-metoxycarbonyl-phenyl)phenyl)-2,4,6-phenylbenzene 9

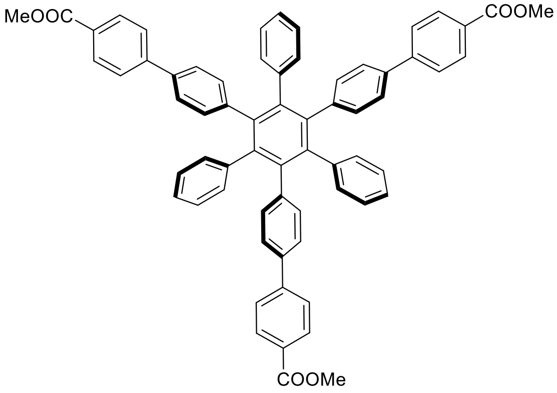

9

To a solution of 1,3,5-(4’-trifluoromethansulphonylphenyl)-2,4,6-phenyl benzene **8** (0.27 g, 0.27 mmol) in dry THF (22 mL), 4-metoxy[illegible]onyl-phenylboronic acid **7** (0.451 g, 2.50 mmol), $Pd(OAc)_2$ (0.003 g, 0.008 mmol), XPhos (0.011 g, 0.02) and K_3PO_4 (0.565 g, 2.50 mmol) were added and placed under inert atmosphere of N_2. The reaction mixture was degassed by N_2 bubbling under sonication for 3 h, then heated at 75 °C for 15 h. The solution was diluted with EtOAc (50 mL), washed with H_2O (3 × 100 mL) and brine (100 mL). The dark organic solution was dried over $MgSO_4$ and concentrated under reduced pressure. The residue was purified by silica gel column chromatography (cyclohexane/EtOAc 7:3) affording **9** as white solids (0.22 g, 83%). MP: 142-145 °C. ^{1}H NMR (300 MHz, CD_3Cl) δ: 8.00 (d, J = 8.7 Hz, 6 H), 7.50 (d, J = 8.7 Hz, 6 H), 7.17 (d, J = 8.5 Hz, 6 H), 6.93 (d, J = 8.4 Hz, 6 H), 6.90-6.86 (m, 15 H), 3.91 (s, 9 H). ^{13}C NMR (100 MHz, CD_3Cl) δ: 167.2, 145.3, 140.8, 140.7, 140.4, 140.1, 136.4, 132.1, 131.5, 130.0, 128.6, 127.0, 126.7, 125.6, 125.5, 52.2. ESI-HRMS: $[M+H]^+$ calc. for $[C_{66}H_{49}O_6]^+$ 937.3529, found 937.3515. IR (solid) ν_{max} (cm^{-1}) 3034, 2947, 1714,1606, 1274, 1004, 738, 700.

S2.9 Synthesis of 1,3,5-tris-(4-(4-carboxylphenyl)phenyl)-2,4,6-phenylbenzene C-L-1 (10)

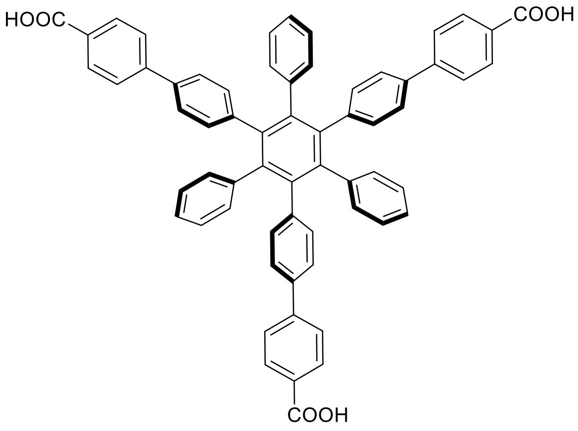

C-L-1

To a solution of **9** (0.22 g, 0.23 mmol) in a mixture 4:1 THF/CH_3OH (35 mL), a solution of NaOH (1 M) was added dropwise until pH: 13-14 was obtained. The reaction mixture was stirred at room temperature for 16 h. Then CH_3OH and THF were removed under reduced pressure and HCl (1 M) was added at 0 °C until pH: 1 was obtained. The white solid was filtered affording **C-L-1** (0.19 g, 90%). MP: >300 °C. ^1H NMR (300 MHz, DMSO) δ: 12.93 (s, 3 H), 7.90 (d, J = 8.3 Hz, 6 H), 7.62 (d, J = 8.3 Hz, 6 H), 7.31 (d, J = 8.3 Hz, 6 H), 7.62 (d, J = 8.3 Hz, 6 H), 7.00-6.79 (m, 15 H). ^{13}C NMR (100 MHz, DMSO) δ: 167.1, 143.3, 140.4, 140.2, 139.9, 139.6, 135.2, 131.7, 131.0, 129.9, 129.4, 126.8, 126.2 125.7, 124.9. IR (solid) ν_{max} (cm^{-1}): 3026, 1604, 1392, 1004, 750, 698. Several mass spectrometric analyses under different conditions and with different ionisation methods were attempted, but in all cases unsatisfactory results were obtained. However, given the NMR spectrometric analysis (above) and the X-ray characterisation results of the resulting MOF, we are confident about the chemical structure of molecule **C-L-1**.

S2.10 Synthesis of [$Zn_4O(CTCP)_6$] C-MOF-1

To a microwave vial (Biotage vial, size L x W x H: 1.5 x 1.5 x 8 cm) **C-L-1** (0.014 g, 0.016 mmol) and $Zn(NO_3)_2 \cdot 6H_2O$ (0.077 g, 0.26 mmol) were added and dissolved in a mixture 1:1 DMF/NMP (4 mL). The reaction mixture was heated at 85°C for 72 h. The crystals obtained were filtered and dried under air affording [$Zn_4O(CTCP)_6$] **C-MOF-1** (0.013 g, 71%). See X-ray crystal data. Solvent exchange procedure: **C-MOF-1** was washed thoroughly with a mixture of 1:1 DMF/NMP (30 mL) to remove any non-coordinated three-carboxyl linker **C-L-1**. In a centrifuge tube, **C-MOF-1** and acetone were added and the suspension was left to soak in the new solvent for 20 min. Subsequently, the suspension was washed with centrifugation (5000 rpm, 30 min) and then the supernatant was carefully removed. This procedure was

applied for 5 cycles. FT-IR (solid) ν_{max} (cm^{-1}): 3354, 3064, 1597, 1394, 767, 659, 566, 533. BET surface area = 509 m^2 g^{-1}; total pore volume of $2.887*10^{-1}$ cc g^{-1}, calculated at $P/P_0 = 0.97674$ from N_2 adsorption at 77 K

S3 Characterization

S3.1 Characterization of BN-L-1

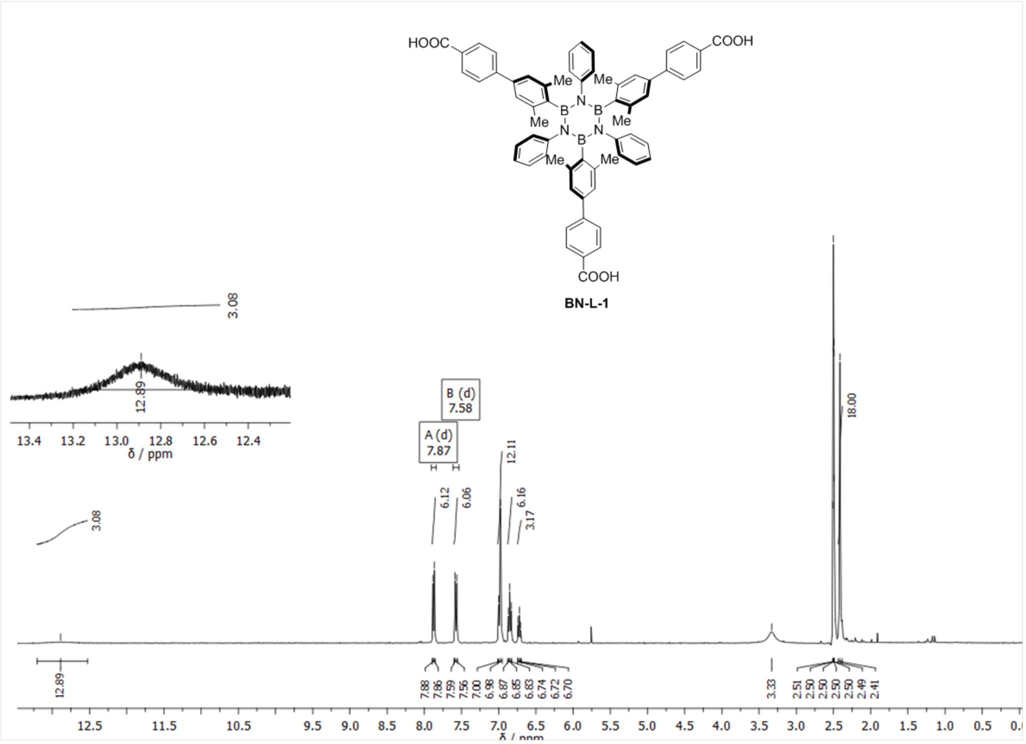

Figure S1. ^{1}H-NMR, 400 MHz, DMSO-*d*6.

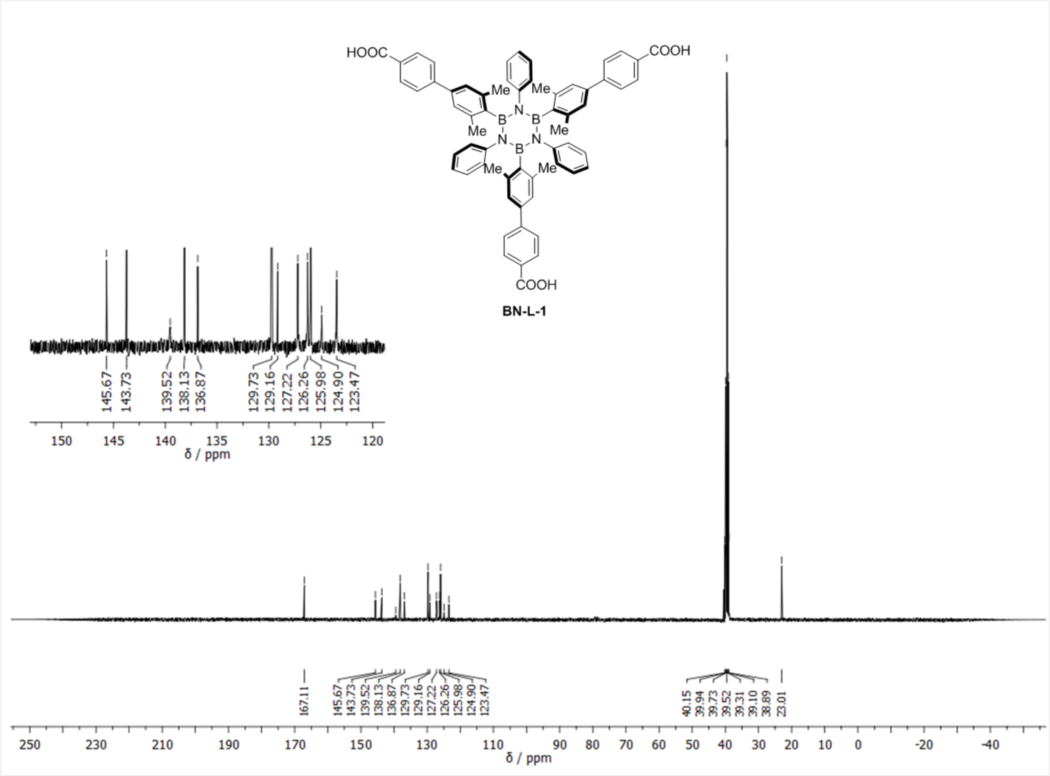

Figure S2. ^{13}C-NMR, 100 MHz, DMSO-*d*6.

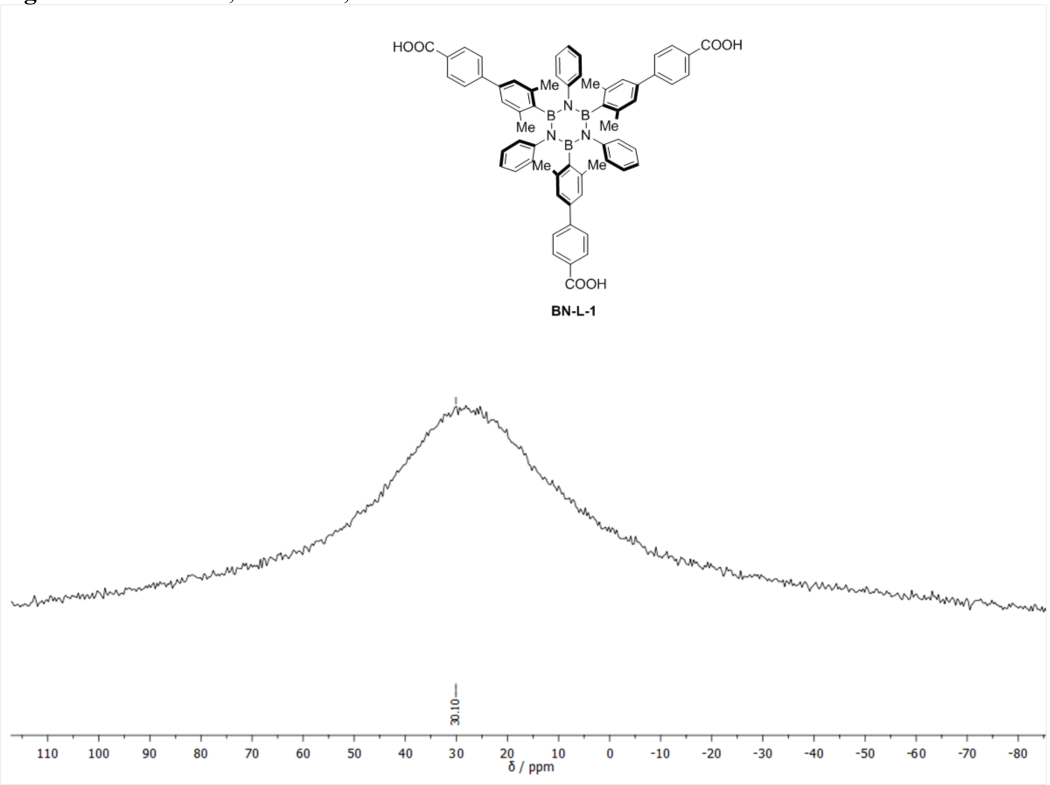

Figure S3. ^{11}B-NMR, 128 MHz, DMSO-*d*6.

S3.2 Characterization of 3b

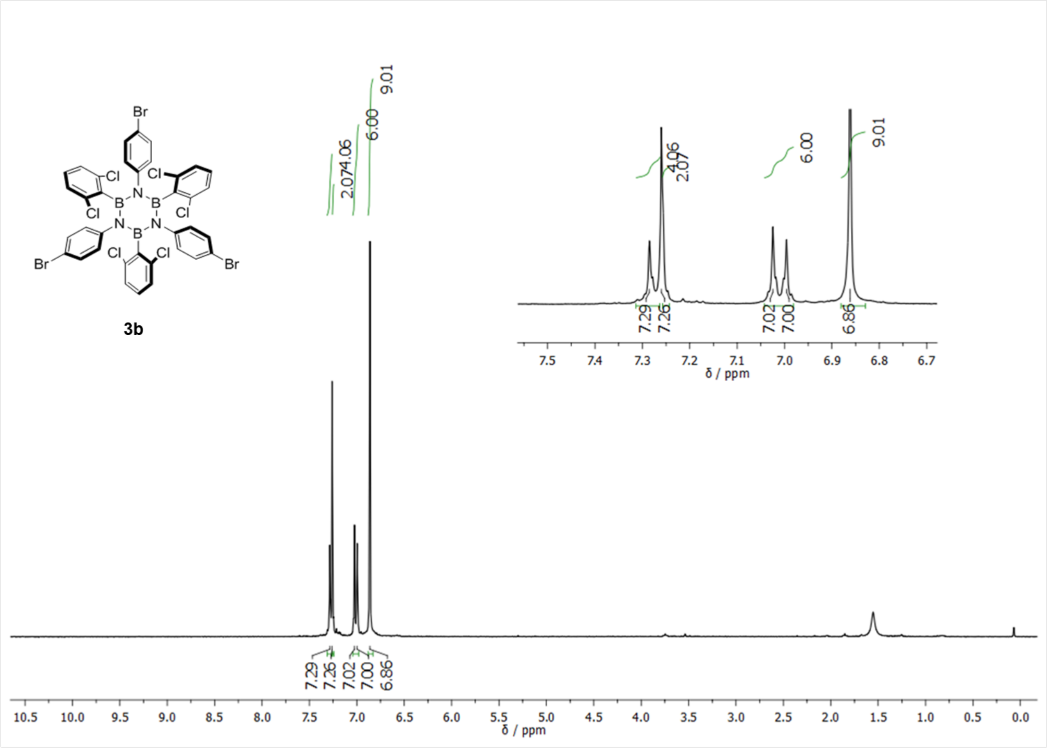

Figure S4. ^{1}H-NMR, 400 MHz, $CDCl_3$.

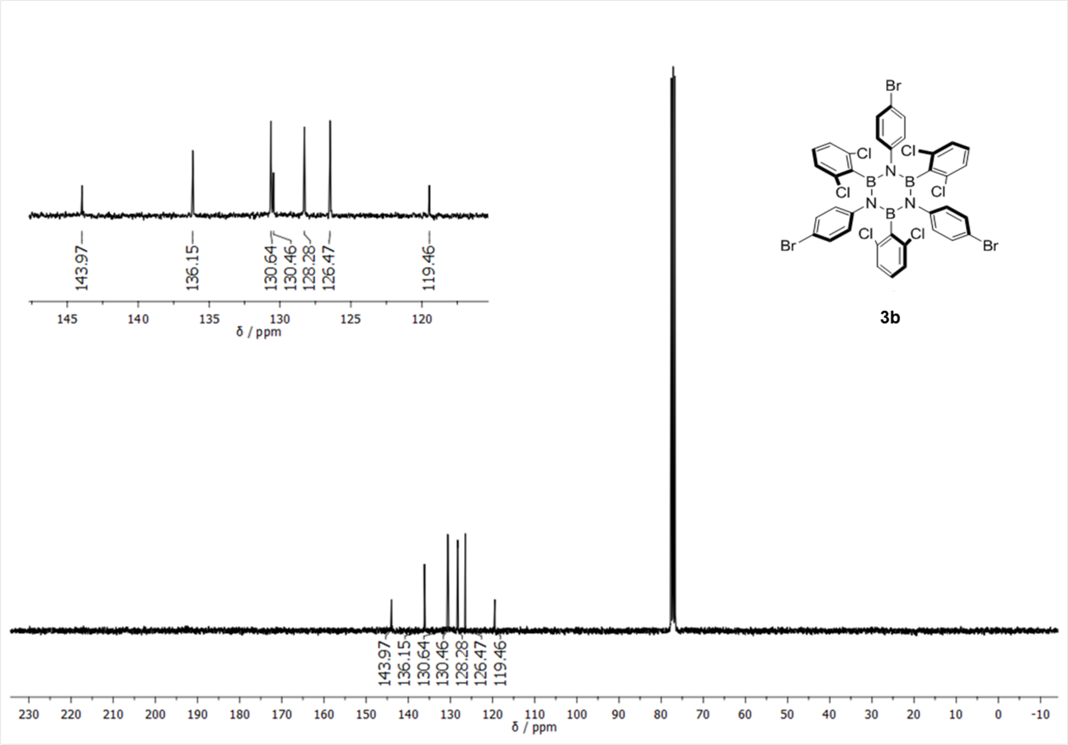

Figure S5. ^{13}C-NMR, 100 MHz, $CDCl_3$.

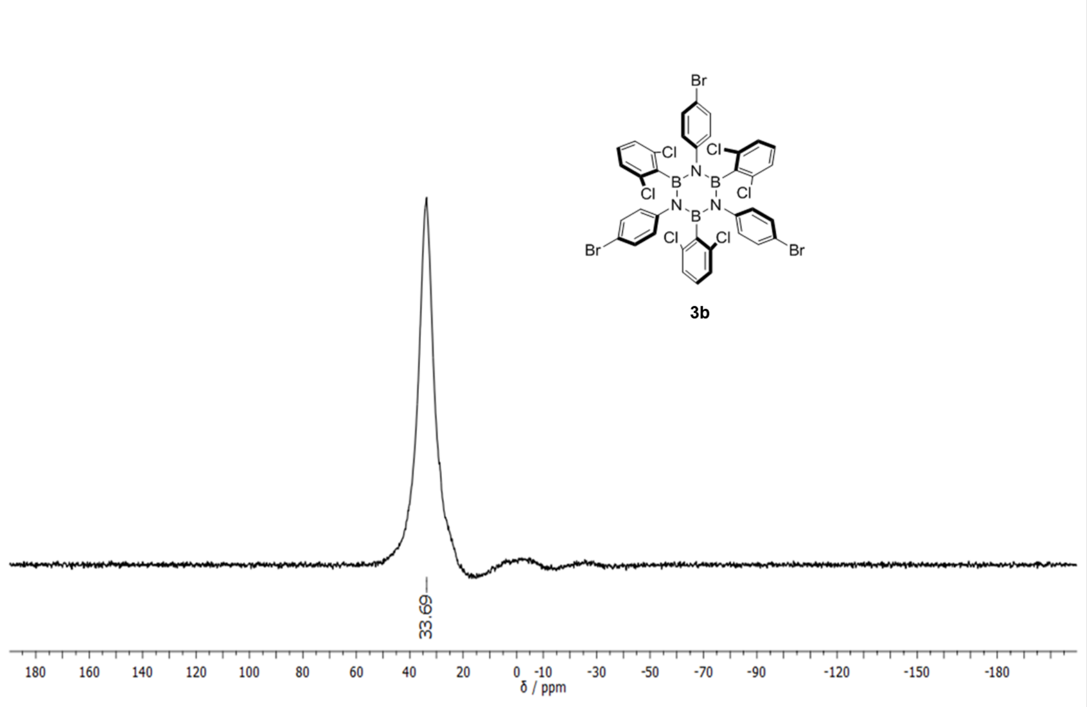

Figure S6. ^{11}B-NMR, 128 MHz, $CDCl_3$.

S3.3 Characterization of 4b

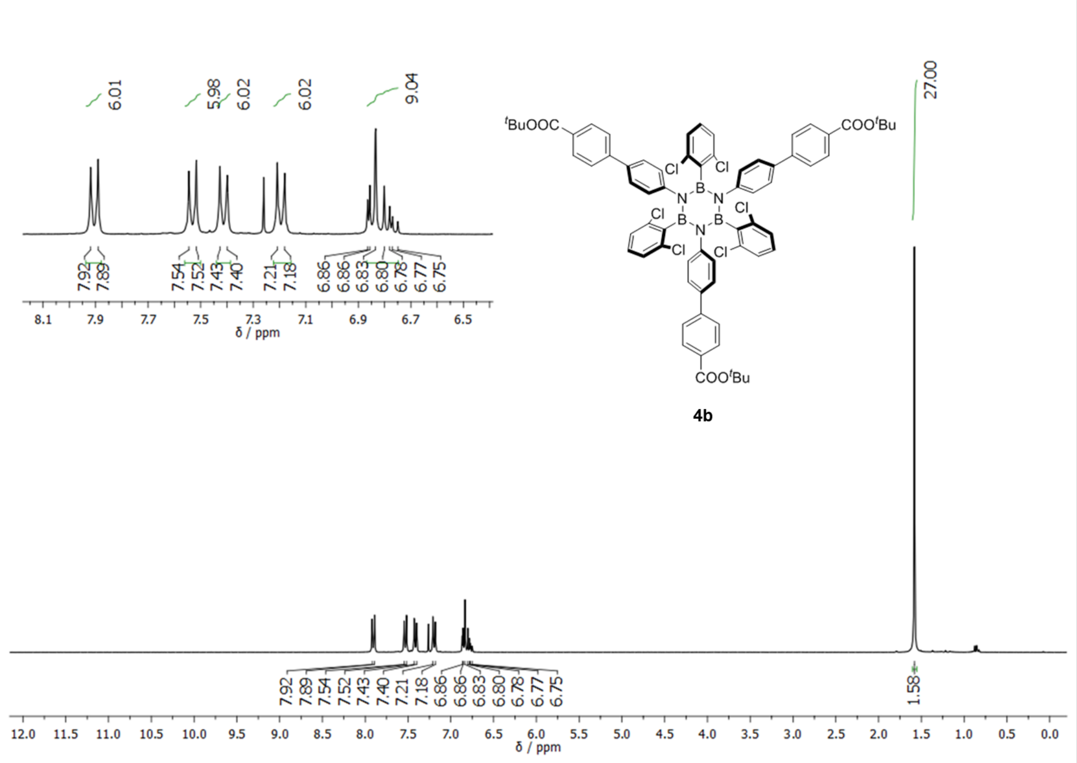

Figure S7. ^{1}H-NMR, 400 MHz, $CDCl_3$.

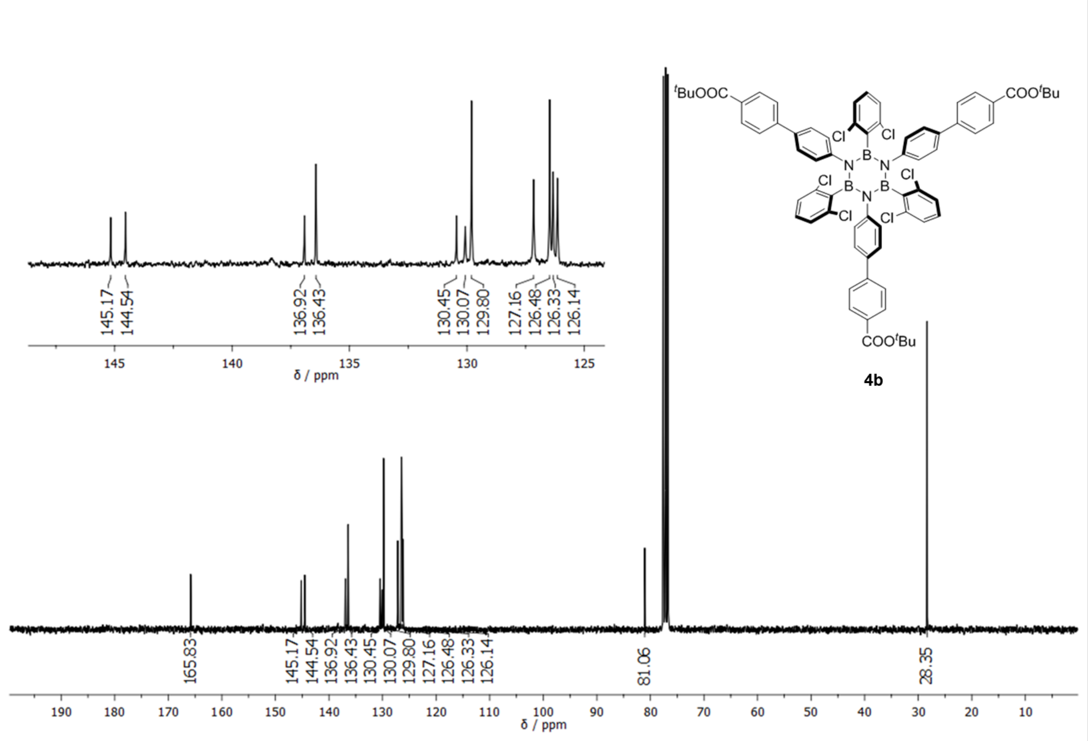

Figure S8. ^{13}C-NMR, 100 MHz, $CDCl_3$.

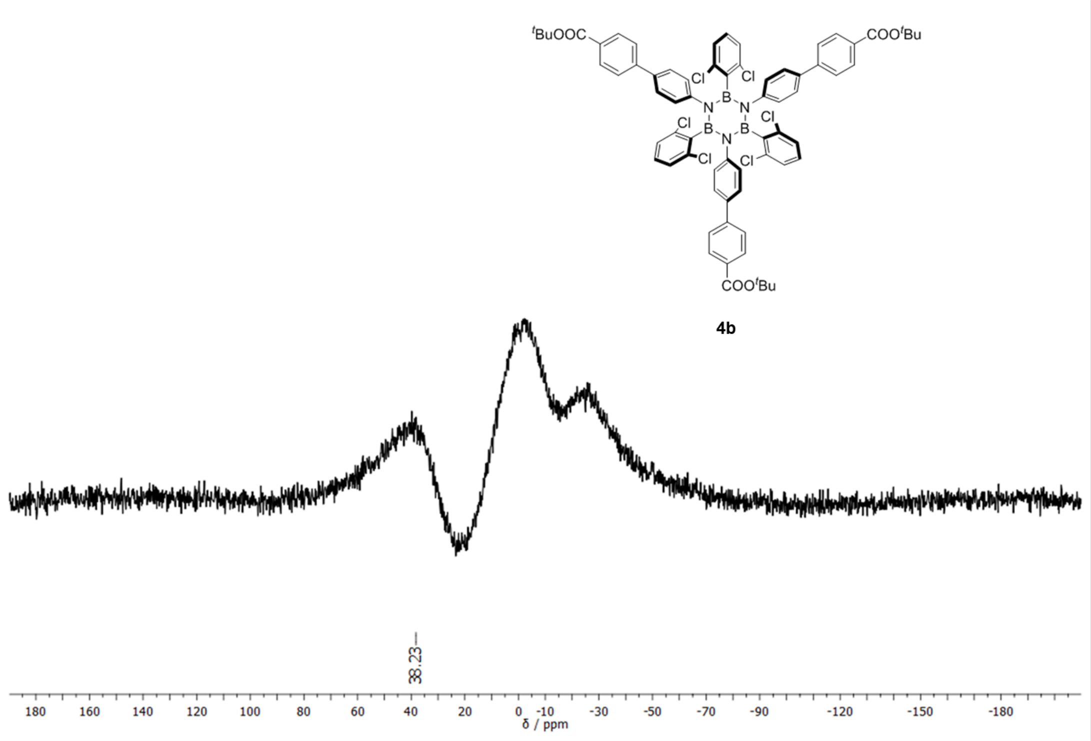

Figure S9. ^{11}B-NMR, 128 MHz, $CDCl_3$ Probe residual signal visible from 20 to -30 ppm.

S3.4 Characterization of BN-L-2

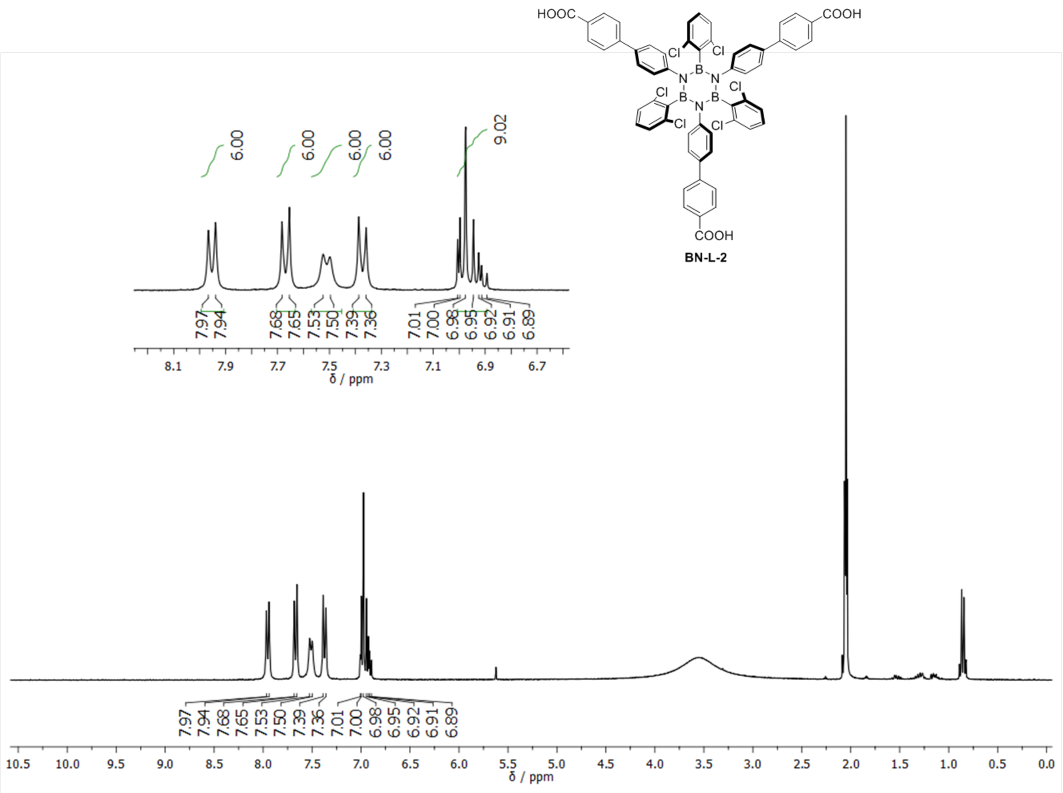

Figure S10. ^{1}H-NMR, 400 MHz, Acetone-*d*6.

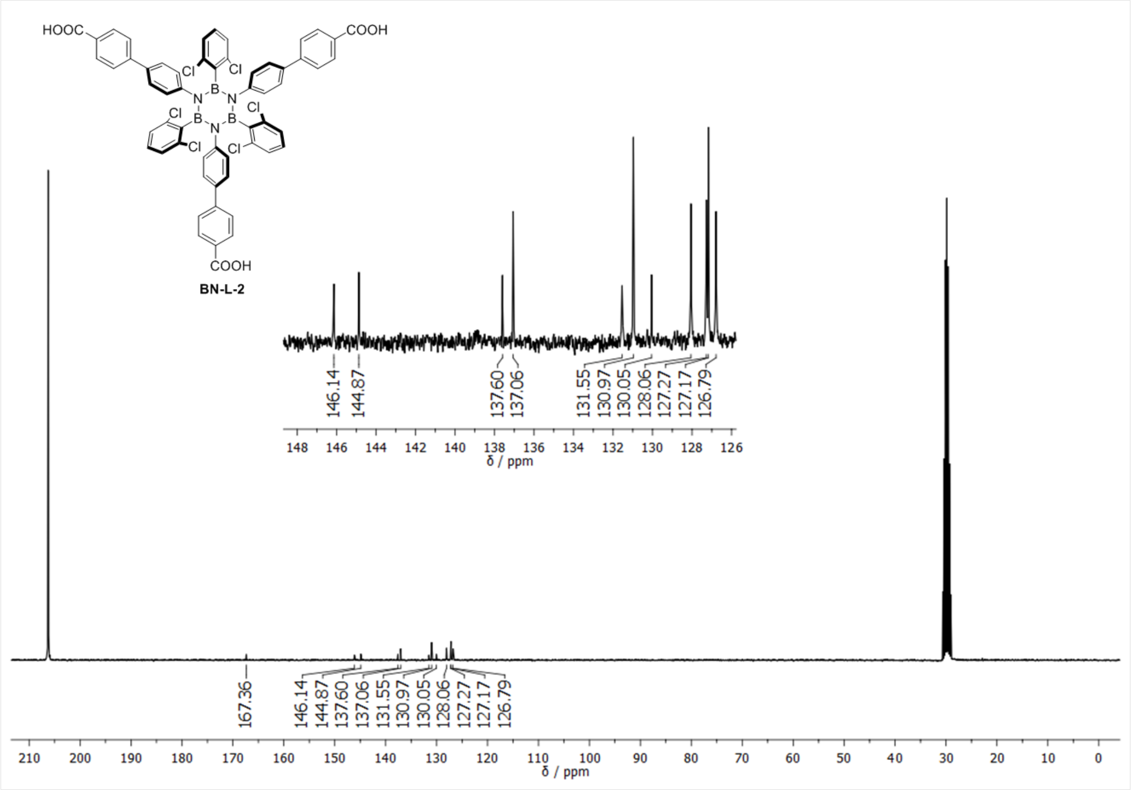

Figure S11. ^{13}C-NMR, 100 MHz, Acetone-*d*6.

S3.5 Characterization of 9

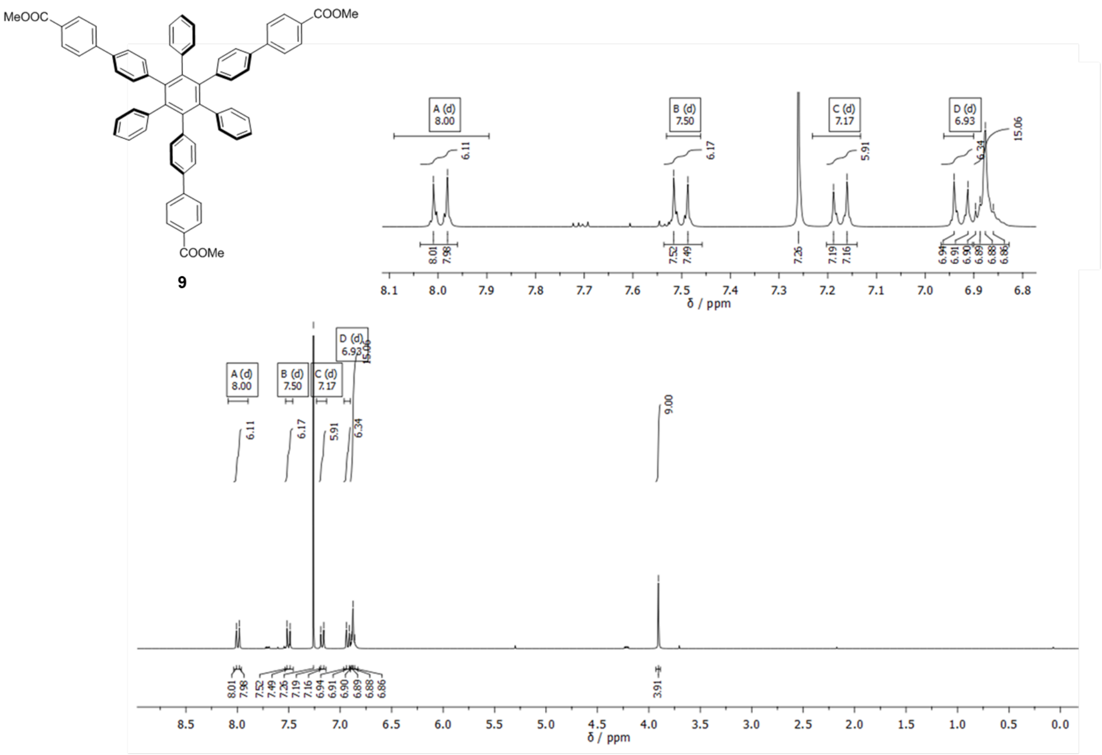

Figure S12. ^{1}H-NMR, 400 MHz, $CDCl_3$.

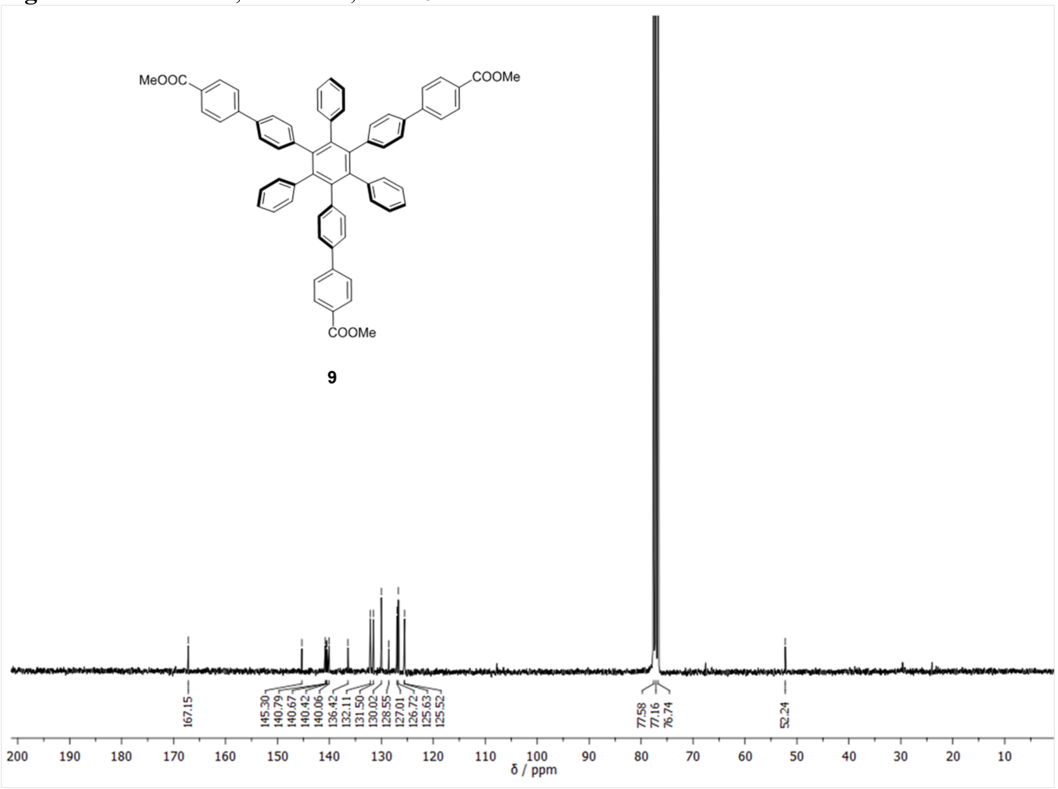

Figure S13. ^{13}C-NMR, 100 MHz, $CDCl_3$.

S3.6 Characterization of C-L-1

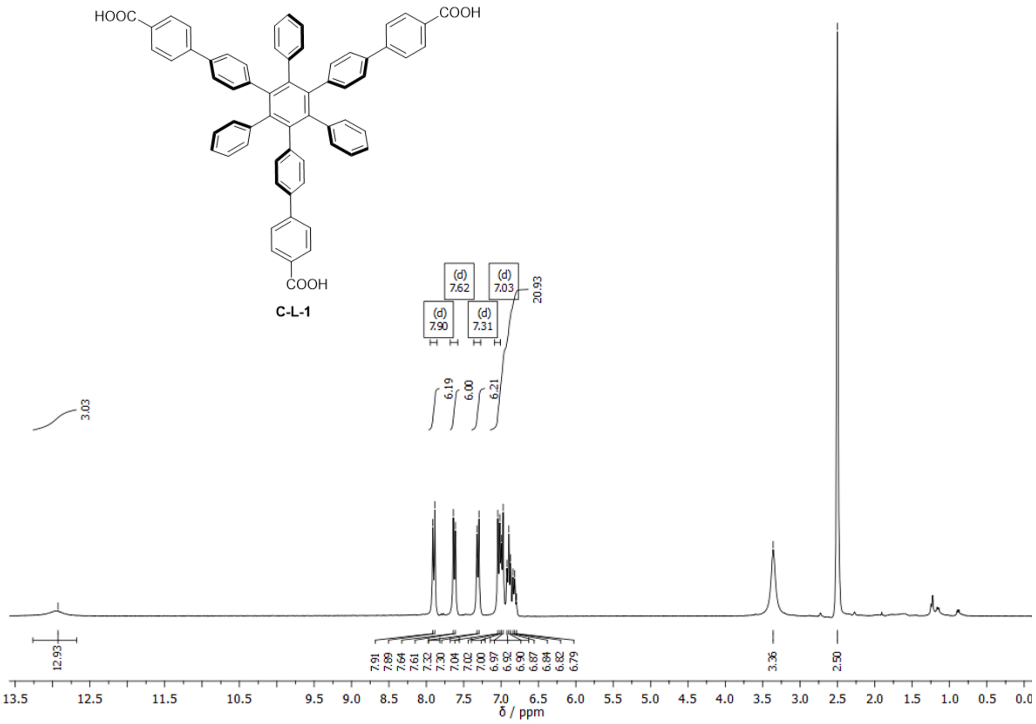

Figure S14. ^{1}H-NMR, 400 MHz, DMSO-*d*6.

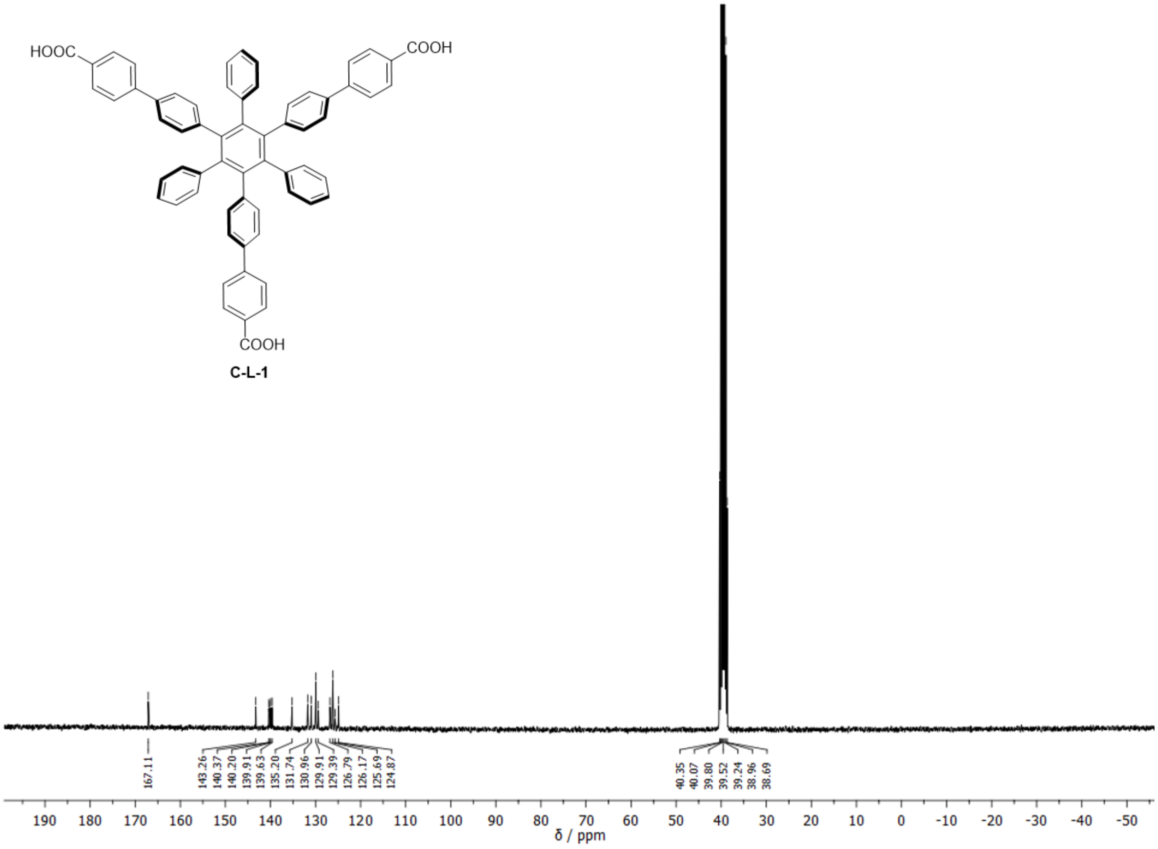

Figure S15. ^{13}C-NMR, 100 MHz, DMSO-*d*6.

S4 Optical Microscope images

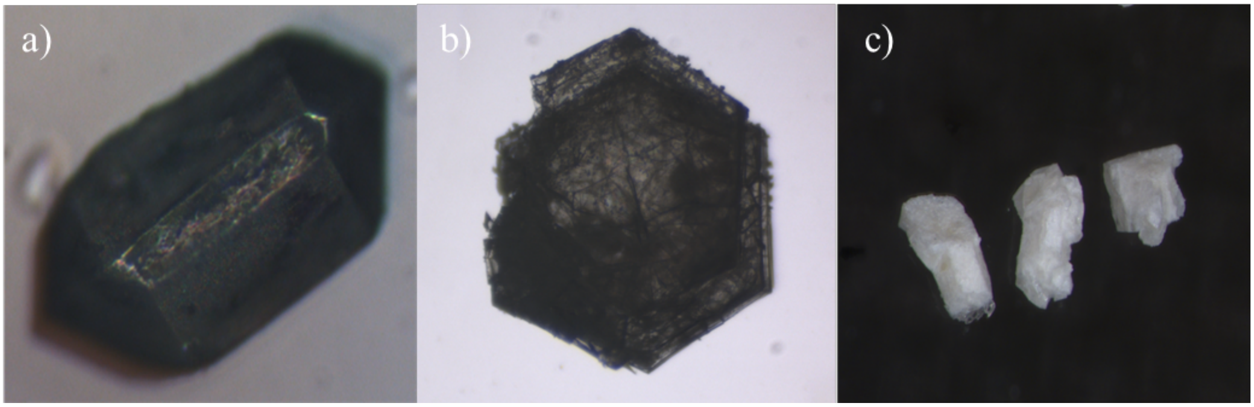

Figure S16. Optical microscope images of a) **BN-MOF-1**; b) **BN-MOF-2;** c) **C-MOF-1**.

S5 Crystallographic data

Crystallographic data for compounds **3b**, **BN-L-1**, **BN-MOF-1**, **BN-MOF-2** and **C-MOF-1** (CCDC: 2038664, 1418729, 2011413, 2011411 and 2011414, respectively) can be obtained free of charge from The Cambridge Crystallographic Data Centre via https://www.ccdc.cam.ac.uk/structures. Data collections were performed at the X-ray diffraction beamline (XRD1) of the Elettra Synchrotron, Trieste (Italy)[2S]. The crystals were dipped in NHV oil (Jena Bioscience, Jena, Germany) and mounted on the goniometer head with kapton loops (MiTeGen, Ithaca, USA). For **BN-MOF-1**, **BN-MOF-2**, and **C-MOF-1**, complete datasets were collected at 250 K and 298 K through the rotating crystal method. Data collection at cryogenic temperatures (100 K) showed significant degradation of diffraction on MOFs and therefore has been applied only to BN-L-1 crystals, whereas the temperature of 200 K was used for 3b . Data were acquired using a monochromatic wavelength of 0.700 Å, on a Pilatus 2M hybrid-pixel area detector (DECTRIS Ltd., Baden-Daettwil, Switzerland). The diffraction data were indexed and integrated using XDS.[3S] Crystals of **BN-MOF-1**, **BN-MOF-2** and **C-MOF-1** were sensitive to radiation damage. Data on larger **C-MOF-1** crystals were corrected for absorption using SADABS code.[4S] The structures were solved by the dual space algorithm implemented in the SHELXT code.[5S] Fourier analysis and refinement were performed by the full-matrix least-squares methods based on F^2 implemented in SHELXL (Version 2018/3)[6S]. The Coot program has been used for modeling.[7S] Anisotropic thermal motion refinement have been applied to all non-solvent atoms. Hydrogen atoms were included at calculated positions with isotropic $U_{factors} = 1.2 \cdot U_{eq}$ or $U_{factors} = 1.5 \cdot U_{eq}$ for methyl and hydroxyl groups (U_{eq} being the equivalent isotropic thermal factor of the bonded non hydrogen atom). Geometric and thermal motion restrains (DFIX, DANG and SIMU) have been applied on disordered moieties. No ordered solvent molecules have been identified in the asymmetric units (ASUs - Figure 1ND) of MOFs; not construable residual density have been removed with the SQUEEZE routine of PLATON[7S] (2852 electrons in 68% - 19182 Å^3 - of BN-MOF-1 unit cell volume, 309 electrons in 5236 Å^3 - 68% of the unit cell of **BN-MOF-2** unit cell volume, 9259 electrons in 63% - 26301 Å^3 - of C-MOF unit cell volume). Crystal form of **BN-L-1** instead show compact molecular packings held by hydrophobic interactions with limited CH···π and π···π contacts. **BN-L-1** bear polar heads that coordinate solvent molecules (DMSO and water for BN-L-1 – Figure S16). Crystals of **BN-MOF-1** show inversion twinning, with a twin fraction of ~50%, identified using PLATON TWINROTMAT algorithm.[8S] Pictures were prepared using Ortep3[9S] and Pymol[10S] software. Essential crystal and refinement data (Table 1S) are reported below. Superimposition of ligand molecule conformations in **BN-L-1** and **BN-MOF-1, C-MOF-1** crystal models show almost equivalent structural arrangements (average coordinates R.M.S.D. 1.06 Å) with larger discrepancies on carboxylic terminals, that are influenced by zinc coordination (Figure S20). Despite the structural similarity of the MOF monomers, two completely different crystal forms have been found for **BN-MOF-1** and **C-MOF-1**. Both structures show two half ligand molecules and half zinc cluster in the

crystallographic asymmetric unit (Figure S19 A and B) but the diverse space group symmetries give raise to two interpenetrated framework in **BN-MOF-1** and three lattices in **C-MOF-1**. MOF packing density is higher in **C-MOF-1** (~35% higher - Table 1S), as a result of a more compact connection between neighbour ligands. Weak hydrophobic interactions, with limited π···π and CH···π contacts are present in both the scaffolds. **BN-MOF-2** crystallize in a centrosymmetric space group with the threefold ligand symmetry perfectly matching the crystallographic ternary axis of *P* -3*m*1 space group (Figure S23). The packing (Figure S24) shows a layered structure with a flat network of ligands bonded by tetrahedral zinc linkers. Ligands on different layers are stacked along crystallographic *c* axis. Adjacent borazine cores are rotated by 60°, to minimize steric repulsions among sidechains and to maximize halogen contacts, as previously reported for chlorinated aromatic molecules.[9S] No ordered solvent molecules could be modeled in the MOF cavities and a remarkable ligand flexibility is shown by liberation of aromatic branches, modeled in two equally populated conformations.

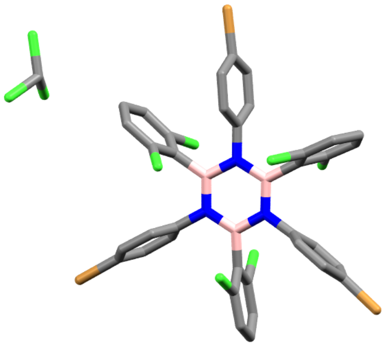

Figure S17. Sticks representation of ASU contents for **3b**. $CHCl_3$ molecules have been found in packing voids.

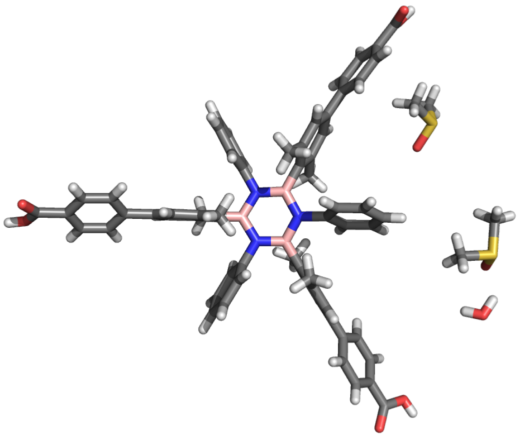

Figure S18. Sticks representation of ASU contents for **BN-L-1**. Solvent molecules have been found in packing voids and are connected to borazine polar tails, through hydrogen bonds.

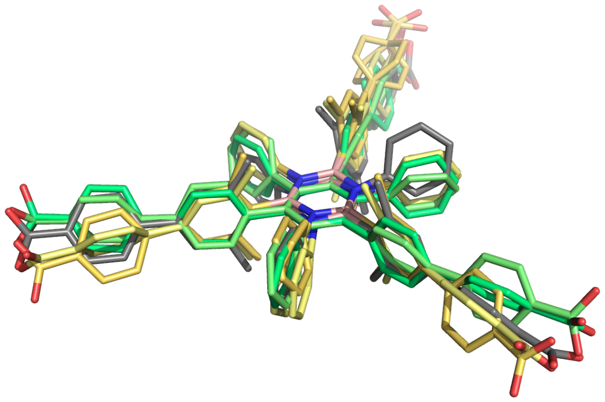

Figure S19. Overlap of **BN-L-1** conformations found in pure ligand structure (grey sticks) and in the two crystallographically independent molecules of **BN-MOF-1** and **C-MOF-1** (yellow and green sticks). Hydrogens omitted for clarity. Average R.M.S.D. of common atoms is 1.06 Å.

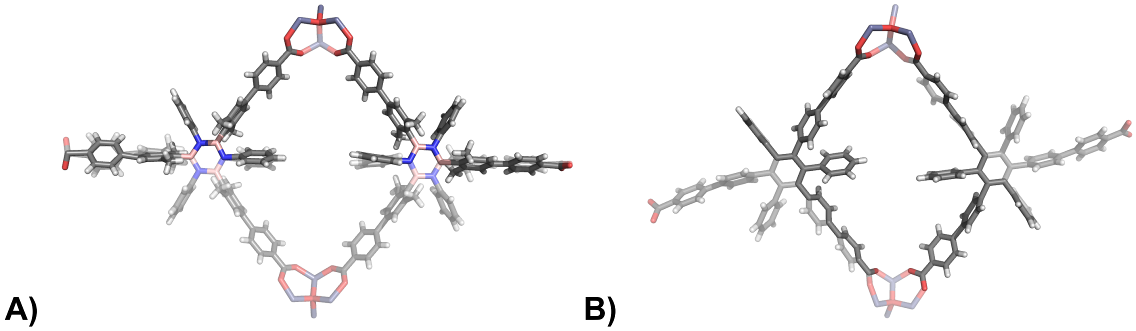

Figure S20. Sticks representation of ASU contents for: A) **BN-MOF-1** and B) C-MOF-1. Two half ligand molecules are crystallographically independent (solid sticks), therefore the missing half ligands (semi transparent sticks) are generated through space group symmetry elements (mirror planes in **BN-MOF-1** and binary axis in **C-MOF-1**).

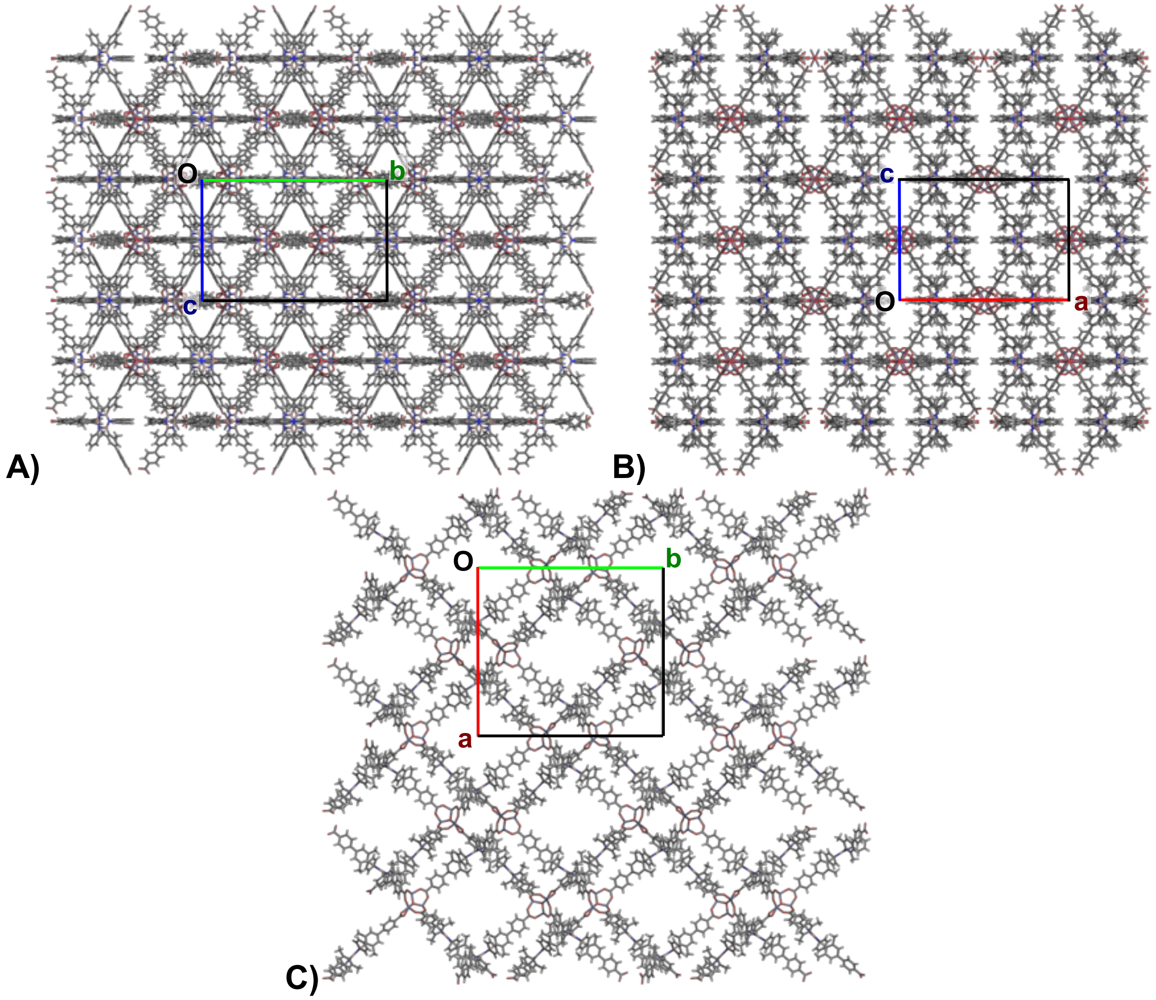

Figure S21. BN-MOF-1 crystal packing views along crystallographic *a*, *b* and *c* axis.

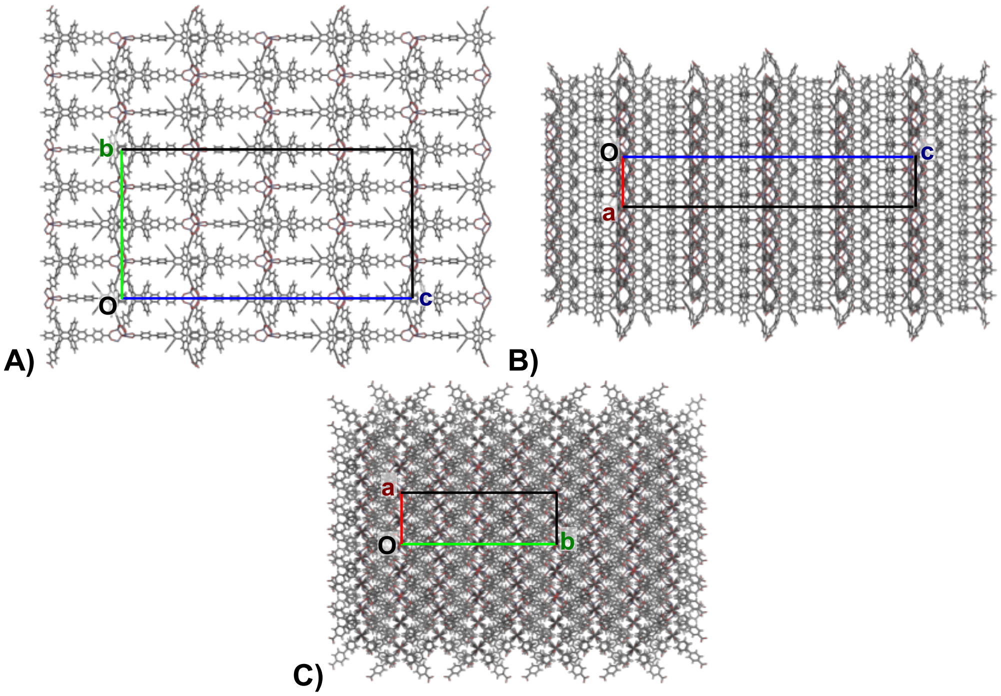

Figure S22. C-MOF-1 crystal packing views along crystallographic *a*, *b* and *c* axis.

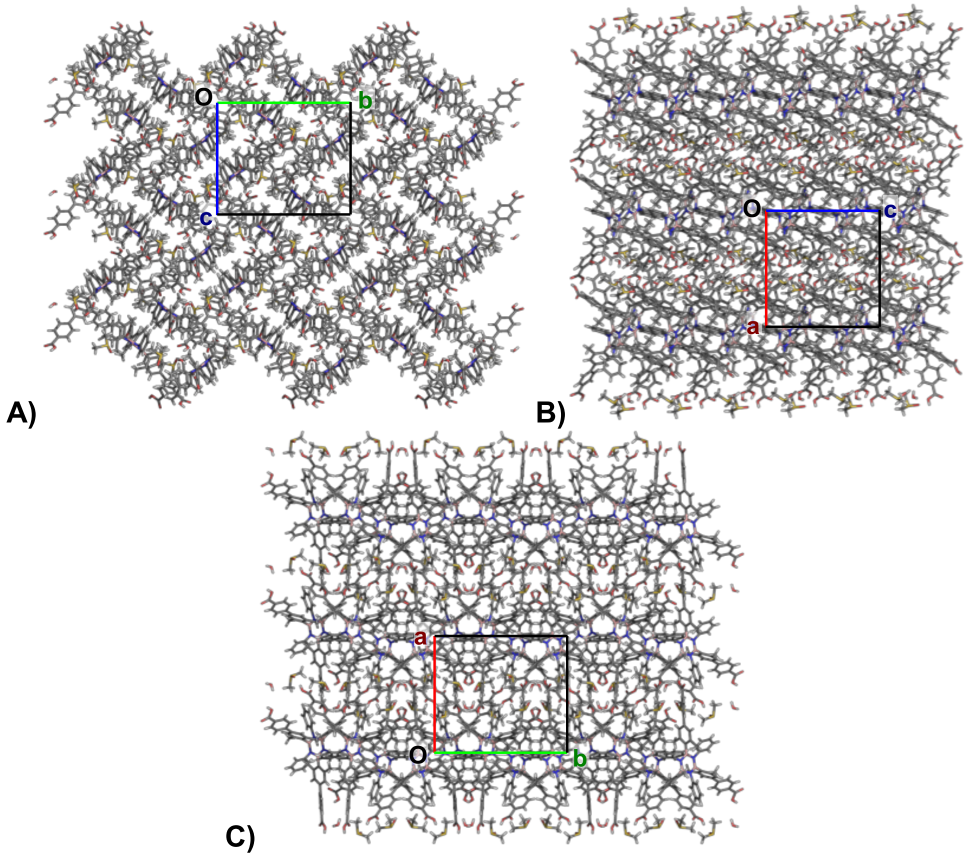

Figure S23. BN-L-1 crystal packing views along crystallographic *a*, *b* and *c* axis.

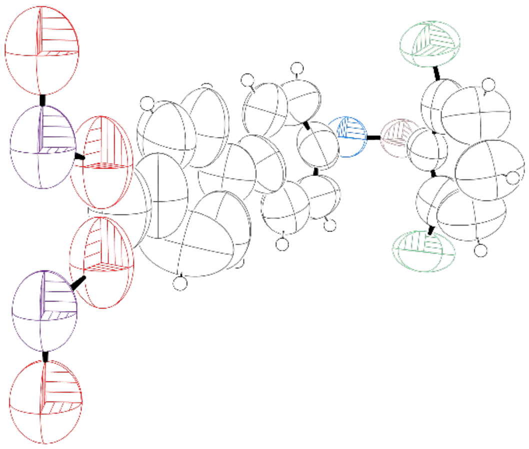

Figure S24. Ellipsoids representation of crystallographic asymmetric unit content (50% probability) for **BN-MOF-2**. One third of the ligand has to be modelled, due to space group symmetry.

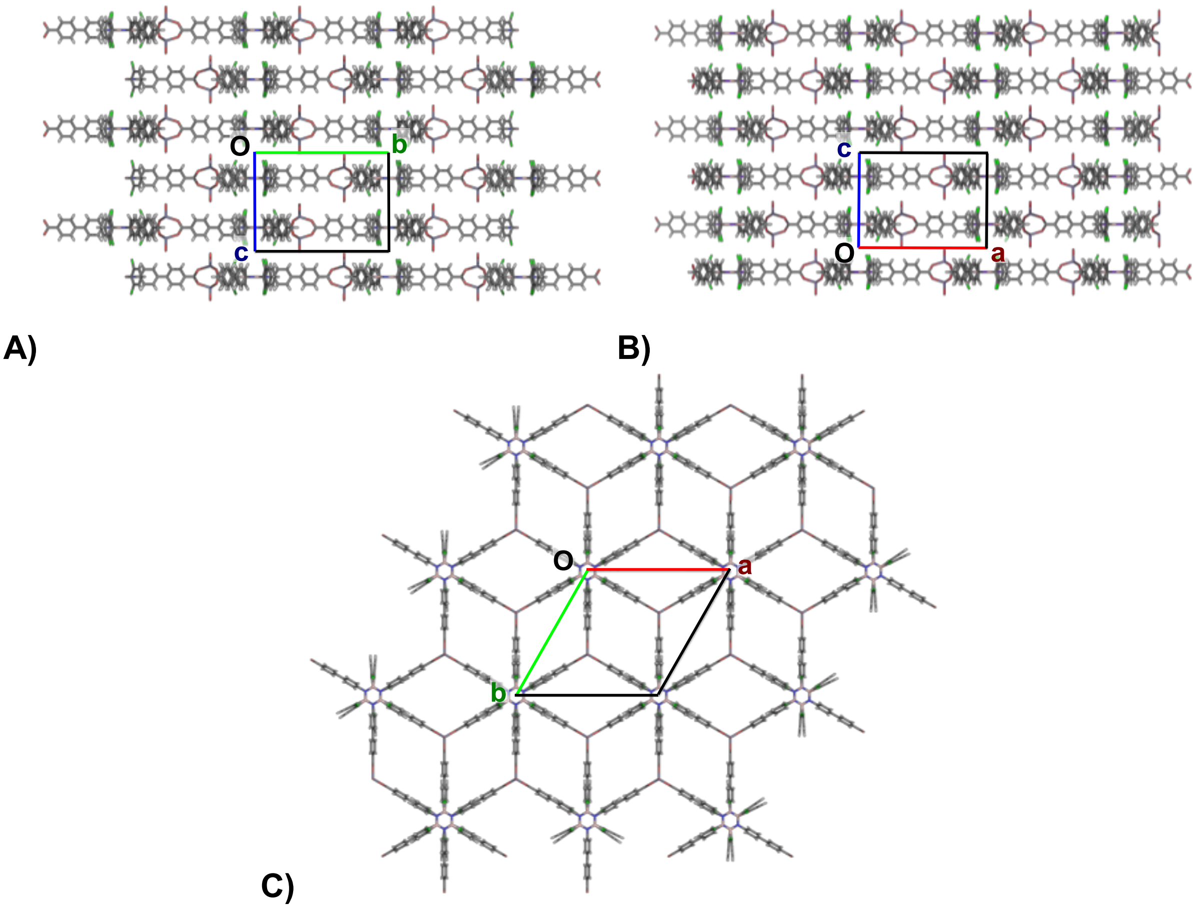

Figure S25. BN-MOF-2 crystal packing views along crystallographic *a*, *b* and *c* axis.

Table 1S. Crystallographic data and refinement details for 3b and BN-L-1.

$R_1 = \Sigma ||Fo|-|Fc|| / \Sigma |Fo|$; $wR_2 = \{\Sigma [w(Fo^2 - Fc^2)^2] / \Sigma [w(Fo^2)^2]\}^{½}$

	**3b**	**BN-L-1**
Chemical Formula	$C_{37}H_{22}B_3Br_3N_3Cl_6$	$C_{67}H_{66.5}B_3N_3O_{8.25}S_2$
Formula weight (g/mol)	1099.78	1142.28
Temperature (K)	200(2)	100(2)
Wavelength (Å)	1.54184	0.700
Crystal system	Monoclinic	Monoclinic
Space Group	$P2_1/n$	$P2_1/c$
Unit cell dimensions	a = 12.6789(4) Å	a = 17.568(4) Å
	b = 20.6834(6) Å	b = 19.981(4) Å
	c = 16.9311(6) Å	c = 16.938(3) Å
	α = 90°	α = 90°
	β = 90.71(3)°	β = 90.71(3)°
	γ = 90°	γ = 90°
Volume (Å^3)	4213.4(3)	5945(2)
Z	4	4
Density (calculated) (g·cm^{-3})	1.734	1.276
Absorption coefficient (mm^{-1})	9.070	0.142
F(000)	2152	2410
Crystal size (mm^3)	0.29 x 0.21 x 0.13	0.10 x 0.05 x 0.05
Crystal habit	Colourless rods	Colourless rods
Theta range for data collection	3.833° to 72.877°	1.14° to 28.17°
Resolution (Å)	0.74	0.74
Index ranges	$-15 \leq h \leq 11$<br>$-17 \leq k \leq 25$<br>$-17 \leq l \leq 20$	$-23 \leq h \leq 23$<br>$-26 \leq k \leq 24$<br>$-22 \leq l \leq 22$
Reflections collected	17704	83392
Independent reflections (data with I>2σ(I))	8193 (9215)	14946 (9215)
Data multiplicity (max resltn)	5.41 (4.65)	5.41 (4.65)
I/σ(I) (max resltn)	8193	11.26 (3.19)
R_{merge} (max resltn)	0.027	0.0688 (0.3337)
Data completeness (max resltn)	99.8%	98.1% (95.5%)
Refinement method	Full-matrix least-squares on F^2	Full-matrix least-squares on F^2
Data / restraints / parameters	8193 / 150 / 533	14946 / 3 / 771
Goodness-of-fit on F^2	1.064	1.059
Δ/σmax		0.001
Final R indices [I>2σ(I)][a]	R_1 = 0.053, wR_2 = 0.1403	R_1 = 0.0848, wR_2 = 0.2034
R indices (all data)[a]	R_1 = 0.0729, wR_2 = 0.1570	R_1 = 0.1422, wR_2 = 0.2455
Largest diff. peak and hole (e·Å^{-3})	1.797 and -1.809	0.882 and -0.834
R.M.S.D. from mean (e·Å^{-3})		0.083

Table 2S. Crystallographic data and refinement details for BN-L-1, BN-MOF-1, C-MOF and BN-MOF-2. $R_1 = \Sigma ||Fo|–|Fc|| / \Sigma |Fo|$; $wR_2 = \{\Sigma [w(Fo^2 – Fc^2)^2] / \Sigma [w(Fo^2)^2]\}^{½}$

	BN-MOF-1	C-MOF-1	BN-MOF-2
Chemical Formula	$C_{93}H_{111}B_3N_{10.5}O_{14}Zn_2$	$C_{111.12}H_{137.62}O_{12.38}N_{18.88}Zn_2$	$C_{70.5}H_{60.5}B_3Cl_6N_{6.5}O_{11.5}Zn_2$
Formula weight (g/mol)	1763.09	2091.45	1558.62
Temperature (K)	250(2)	298(2)	298(2)
Wavelength (Å)	0.700	0.700	0.700
Crystal system	Orthorhombic	Orthorhombic	Trigonal
Space Group	*Pnnm*	*Fdd*2	*P*-3*m*1
Unit cell dimensions	a = 33.013(7) Å	a = 13.488(3) Å	a = 23.999(3) Å
	b = 36.291(7) Å	b = 40.043(8) Å	b = 23.999(3) Å
	c = 23.658(5) Å	c = 77.416(15) Å	c = 15.438(4) Å
	α = 90°	α = 90°	α = 90°
	β = 90°	β = 90°	β = 90°
	γ = 90°	γ = 90°	γ = 120°
Volume (Å^3)	28344(10)	41812(14)	7700(3)
Z	8	16	2
Density (calculated) (g·cm^{-3})	0.826	1.329	0.672
Absorption coefficient (mm^{-1})	0.343	0.475	0.415
F(000)	7436	17728	1596
Crystal size (mm^3)	0.10 x 0.05 x 0.05	0.15 x 0.10 x 0.10	0.10 x 0.02 x 0.02
Crystal habit	Colourless rods	Colourless rods	Colourless thin rods
Theta range for data collection	1.01° to 24.62°	1.04° to 29.98°	1.30° to 21.37°
Resolution (Å)	0.84	0.70	0.96
Index ranges	$-36 \leq h \leq 39$<br>$-43 \leq k \leq 40$<br>$-26 \leq l \leq 26$	$-19 \leq h \leq 13$<br>$-57 \leq k \leq 57$<br>$-105 \leq l \leq 110$	$-24 \leq h \leq 24$<br>$-24 \leq k \leq 23$<br>$-26 \leq l \leq 15$
Reflections collected	90793	57900	29225
Independent reflections (data with I>2σ(I))	24725 (14888)	27821 (19722)	3235 (2251)
Data multiplicity (max resltn)	3.47 (3.46)	3.54 (2.58)	8.98 (9.30)
I/σ(I) (max resltn)	11.62 (1.55)	16.58 (1.93)	10.60 (1.67)
R_{merge} (max resltn)	0.0414 (0.6314)	0.0418 (0.4175)	0.1106 (0.7254)
Data completeness (max resltn)	96.0% (93.9%)	94.0% (84.0%)	99.5% (98.9%)
Refinement method	Full-matrix least-squares on F^2	Full-matrix least-squares on F^2	Full-matrix least-squares on F^2
Data / restraints / parameters	24725 / 274 / 773	27821 / 74 / 656	3235 / 96 / 188
Goodness-of-fit on F^2	1.051	0.995	1.043
Δ/σmax	0.007	0.005	0.001
Final R indices [I>2σ(I)][a]	R_1 = 0.0986, wR_2 = 0.2226	R_1 = 0.0516, wR_2 = 0.1472	R_1 = 0.1260, wR_2 = 0.2543
R indices (all data)[a]	R_1 = 0.1231, wR_2 = 0.2359	R_1 = 0.0653, wR_2 = 0.1625	R_1 = 0.1445, wR_2 = 0.2673
Largest diff. peak and hole (e·Å^{-3})	0.470 and -0.545	0.291 and -0.503	0.544 and -0.275
R.M.S.D. from mean (e·Å^{-3})	0.059	0.042	0.039

S6 Thermal Gravimetric analysis

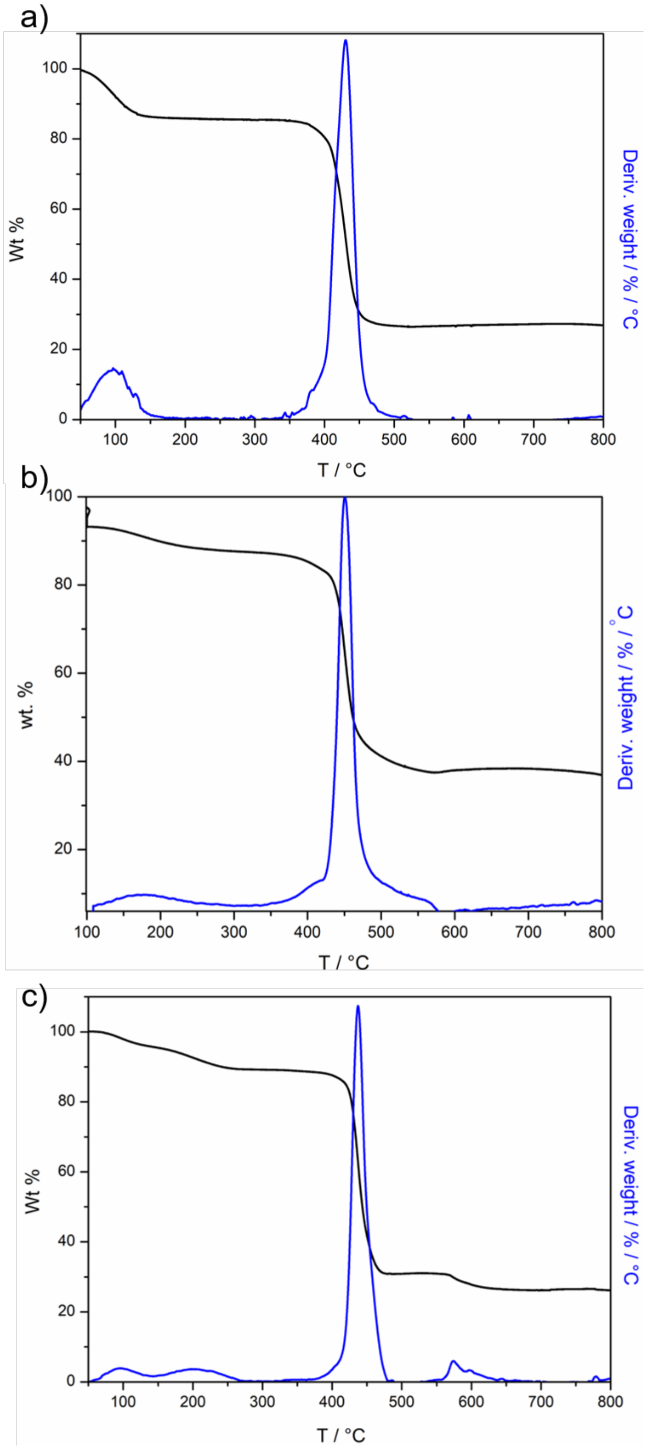

Figure S26. TGA curves (black) and corresponding DTG curves (blue) of a) **BN-MOF-1**; b) **BN-MOF-2**; c) **C-MOF-1**

S7 Powder X-Ray Diffraction

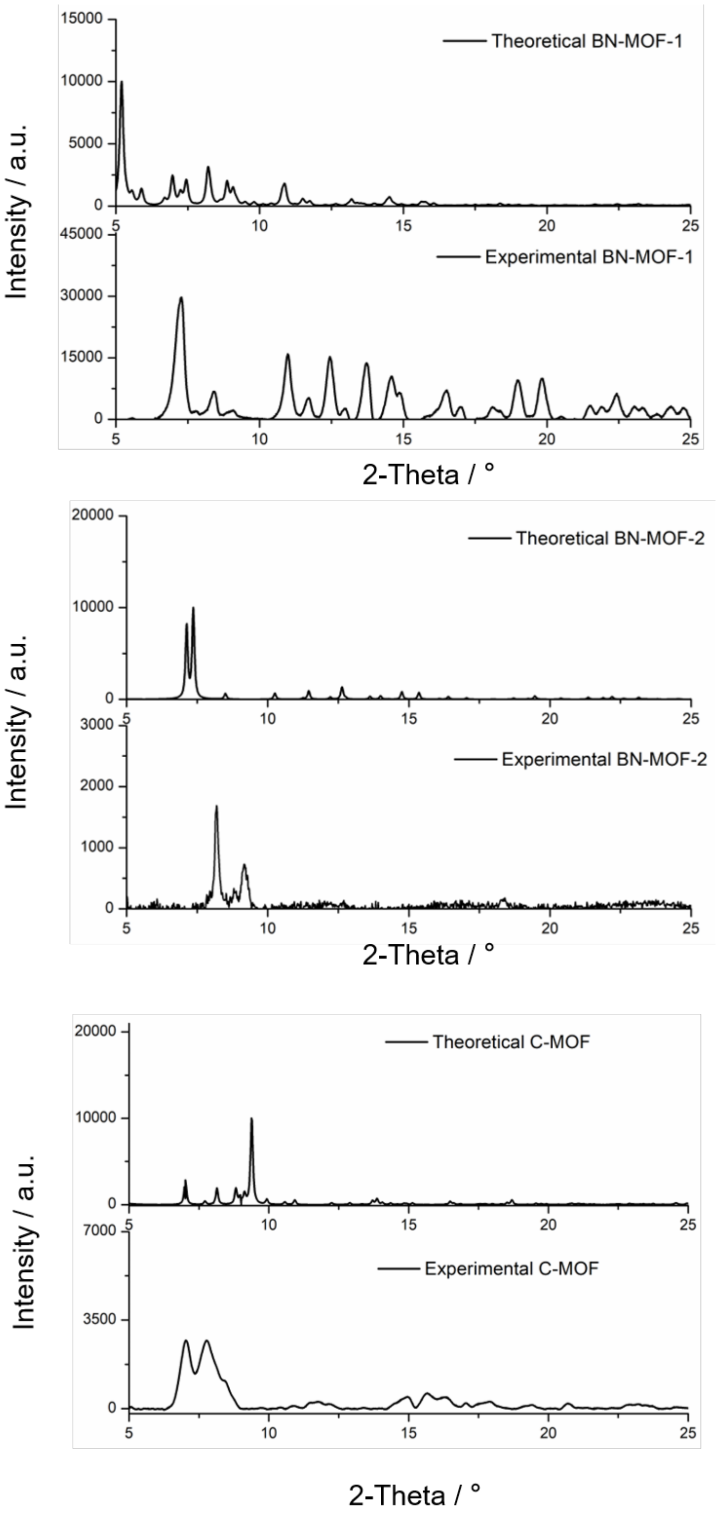

Figure S27. Theoretical and experimental PXRD spectra of **BN-MOF-1**, **BN-MOF-2** and **C-MOF-1.**

S8 Linkers and MOFs Infrared spectra

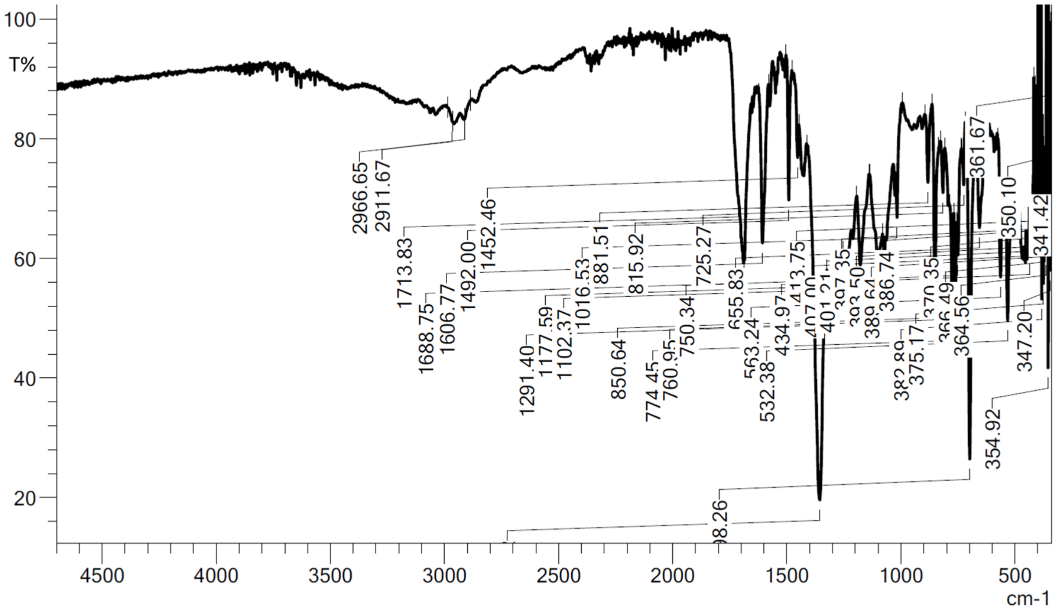

Figure S28. ATR/FT-IR spectrum of **BN-L-1.**

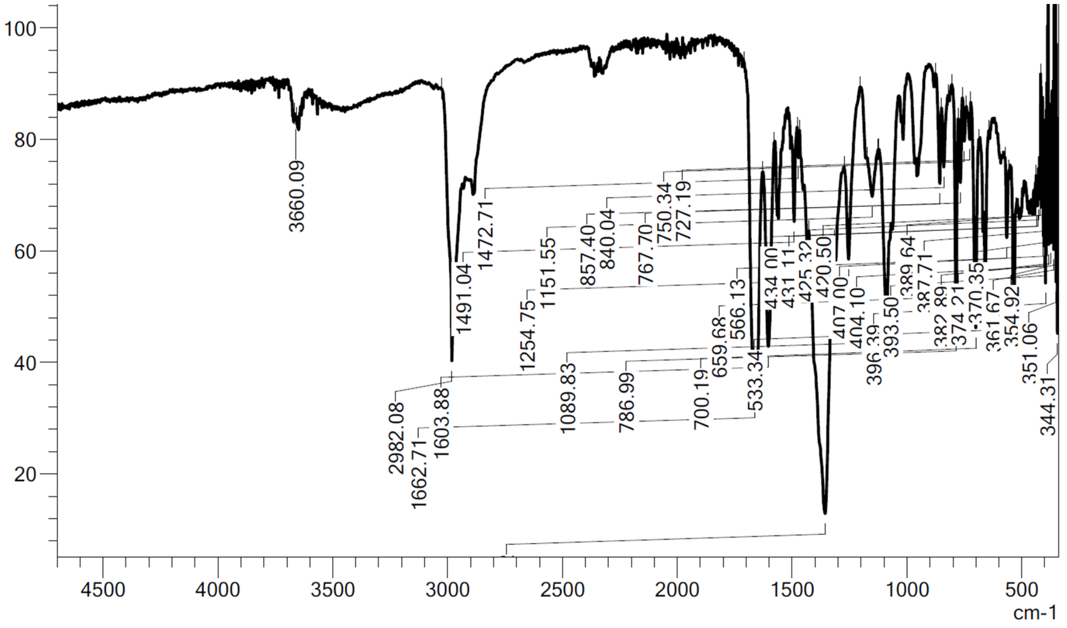

Figure S29. As synthesised **BN-MOF-1** ATR/FT-IR spectrum.

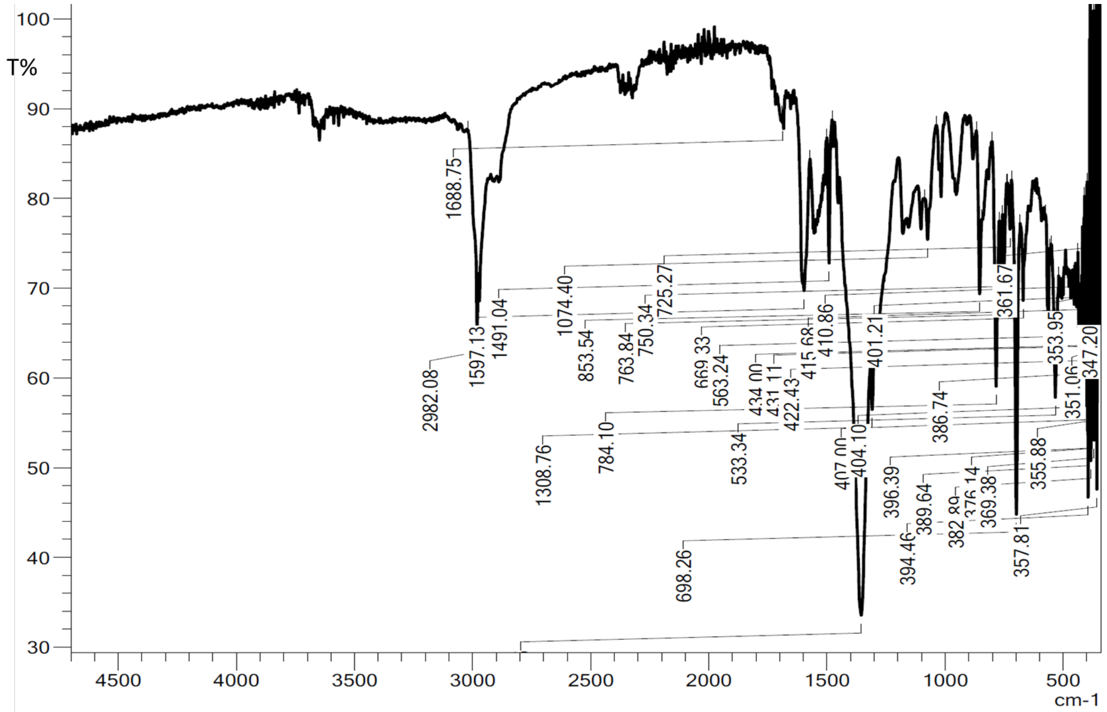

Figure S30. Activated **BN-MOF-1** ATR/FT-IR spectrum.

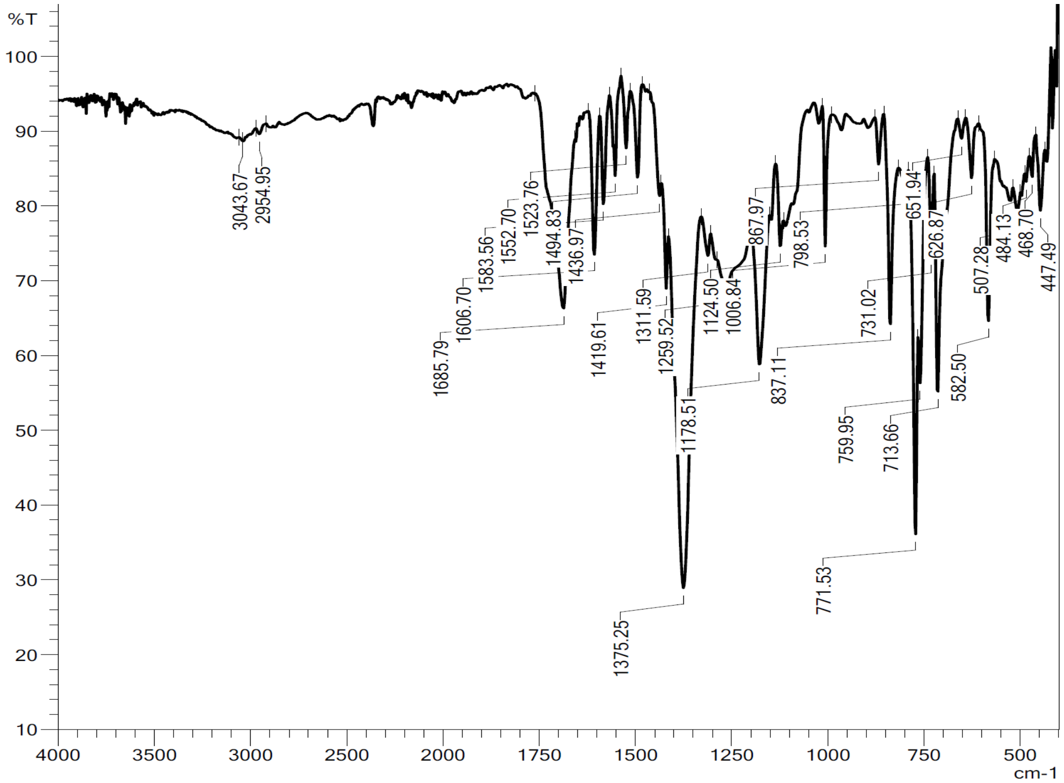

Figure S31. ATR/FT-IR spectrum of **BN-L-2.**

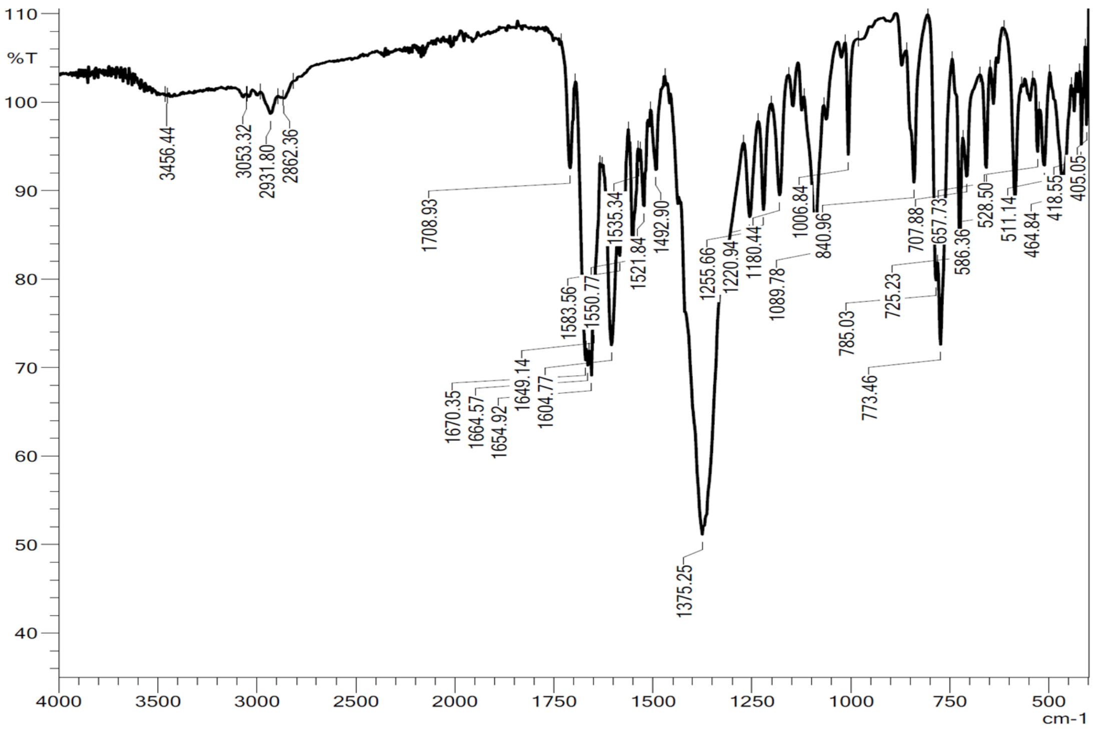

Figure S32. As synthesised **BN-MOF-2** ATR/FT-IR spectrum.

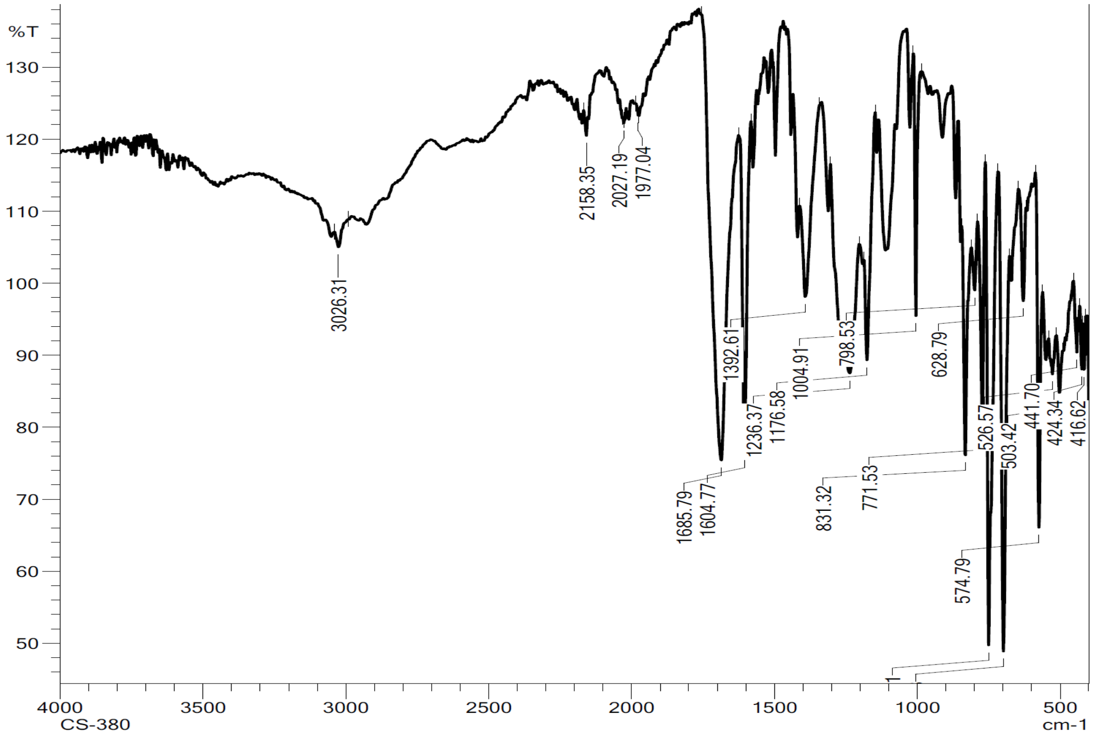

Figure S33. ATR/FT-IR spectrum of **C-L-1.**

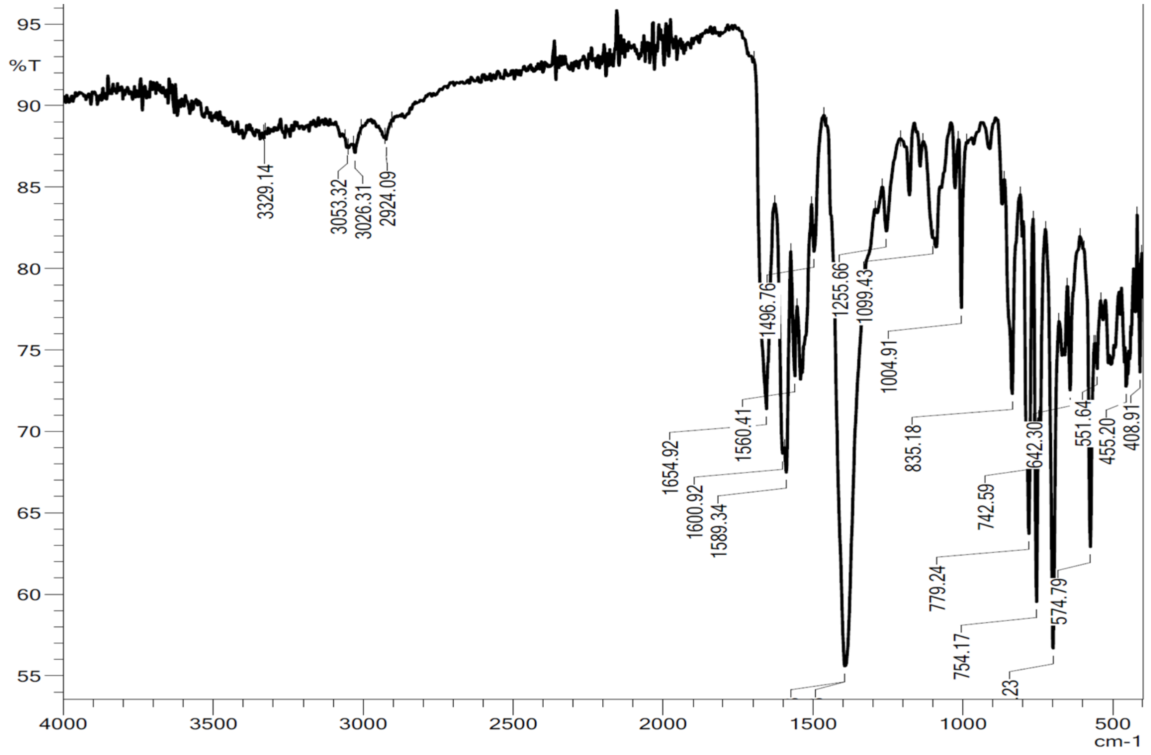

Figure S34. As synthesised **C-MOF-1** ATR/FT-IR spectrum.

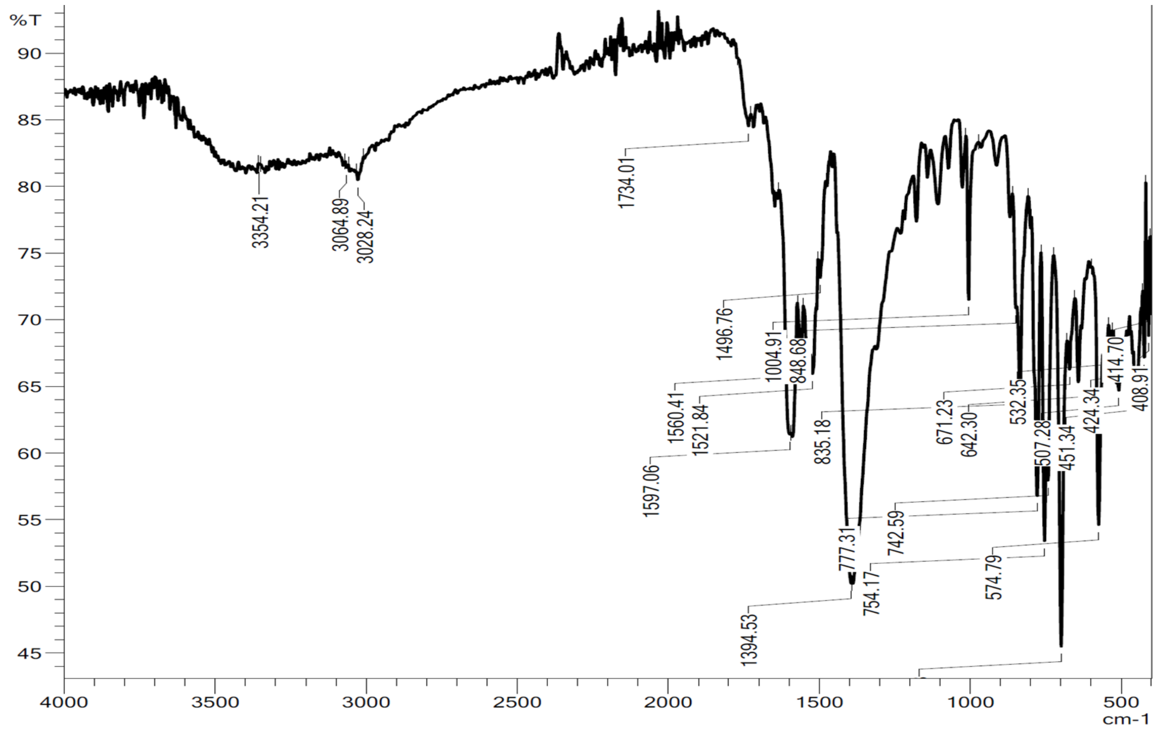

Figure S35. Activated **C-MOF-1** ATR/FT-IR spectrum.

S9 Scanning electron microscope images of activated BN-MOF-2

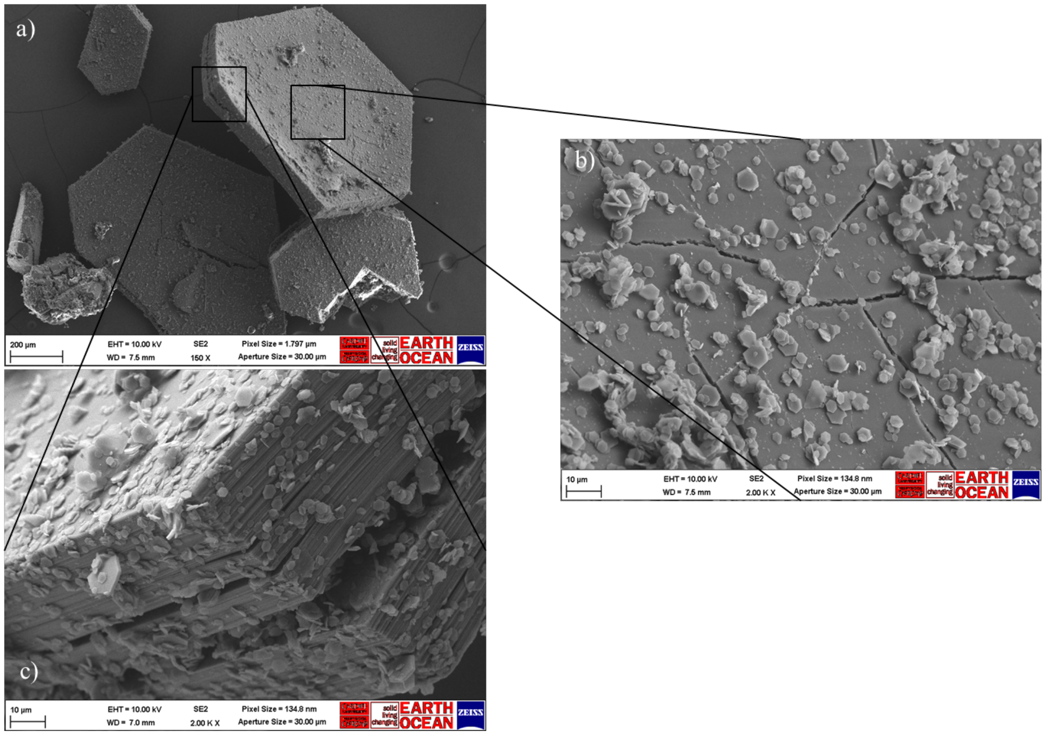

Figure S36. Activated **BN-MOF-2** SEM images. b) and c) zoomed in images on the indicated areas in image a).

S10 Pores size distribution

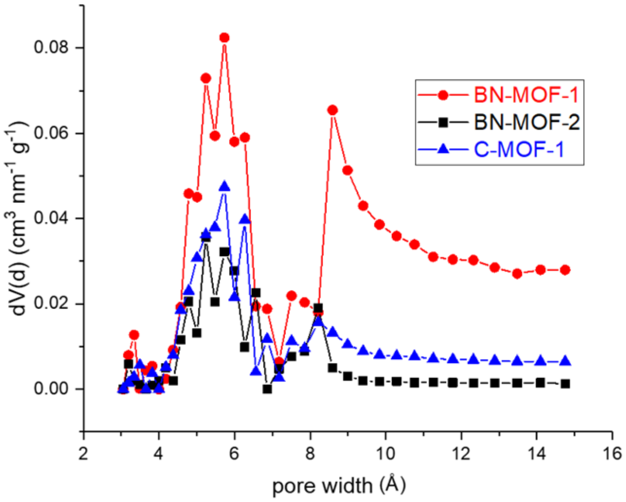

Figure S37. DFTL pore size distribution for **BN-MOF-1**, **BN-MOF-2** and **C-MOF-1** calculated from CO_2 adsorption isotherms at 273 K.

S11 References

1S. D. Marinelli, F. Fasano, B. Najjari, N. Demitri and D. Bonifazi *J. Am. Chem. Soc.* **2017**, *139*, 5503-5519.

2S. Lausi A., Polentarutti M., Onesti S., Plaisier J. R., Busetto E., Bais G., Barba L., Cassetta A., Campi G., Lamba D., Pifferi A., Mande S. C., Sarma D. D., Sharma S. M., Paolucci G. *The European Physical Journal Plus*, **2015**, BNL(*43*), 1-8.

3S. Kabsch W. XDS. *Acta Crystallographica Section D*, **2010**, *66* (2), 125-132.

4S. Sheldrick G. M. (**2012**). SADABS. University of Göttingen, Germany.

5S. Sheldrick G. M., *Acta Crystallographica Section A* **2015**, *71*, 3-8.

6S. Sheldrick G. M., *Acta Crystallographica Section C* **2015**, *71*, 3-8.

7S. Emsley P., Lohkamp, B., Scott W. G., Cowtan K., *Acta Crystallographica Section D* **2010**, *66*(4), 486-501.

8S. Spek A. *Acta Crystallographica Section C*, **2015**, *71*(1), 9-18.

9S. Farrugia L. *Journal of Applied Crystallography*, **2012**, *45*(4), 849-854.

10S. L. Schrodinger **2015** The PyMOL Molecular Graphics System. Schrodinger, LLC. http://www.pymol.org.
